# A New Approach to Synthesize of 4-Phenacylideneflavene Derivatives and to Evaluate Their Cytotoxic Effects on HepG2 Cell Line

**DOI:** 10.3390/molecules22081296

**Published:** 2017-08-09

**Authors:** Hongbin Chen, Yang Xu, Yinan Zhang, Zongping Zheng

**Affiliations:** 1 Fujian Province Key Laboratory for the Development of Bioactive Material from Marine Alge, College of Oceanology and Food Science, Quanzhou Normal University, Quanzhou 362000, China; chenhongbin@qztc.edu.cn; 2 School of Pharmaceutical Sciences, Xiamen University, Xiamen 361005, China; xu_yang@xmu.edu.cn; 3 College of pharmacy, University of Kentucky, Lexington, KY 40536, USA; noahzhang@gmail.com

**Keywords:** substituted acetophenones, 2,4-dihydroxybenzaldehyde, 4-phenacylideneflavenes, cytotoxic effect, HepG2 cell line

## Abstract

In this study, a convenient approach and green procedure for the synthesis of 4-phenacylideneflavenes has been developed from the reaction between 2,4-dihydroxybenzaldehyde and substituted acetophenones using boric acid as a catalyst in polyethylene glycol 400. Seven 4-phenacylideneflavenes were synthetized and their structures were confirmed by NMR and mass spectral analyses. Meanwhile, their possible mechanism of formation was also discussed. These products were found to have potential cytotoxic effect on HepG2 cell line with IC_50_ values from 12.5 to 50 µM.

## 1. Introduction

Chromene moieties are widely found in flavonoid derivatives. Their derivatives have become of much interest due to a variety of biological activities such as anticancer, antimicrobial, anti-inflammatory, and anti-HIV activities [1,2,3,4]. 4-Phenacylideneflavene derivatives are one type of chromene moiety derivatives. However, the methods for the synthesis of phenacylideneflavene derivatives were limited to only a few until now. Bhattacharjee and co-author recently reported one-pot synthesis of 4-phenacylideneflavene derivatives with a simple reaction procedure, high bond-forming efficiency, good yields, and environmentally benign reaction conditions using bromodimethylsulfonium bromide as catalyst in [5]. Sashidhara et al. also reported one-pot synthesis of 4-phenacylideneflavene derivatives through the reaction of salicylaldehydes and acetophenones using I_2_ as catalyst under reflux conditions [6]. Other methods available for the synthesis of phenacylideneflavene derivatives were as follows: Fichtner et al. reported to obtain 4-phenacylideneflavene derivatives from the reaction of flavylium salt with 1-phenyl-1-(trimethylsiloxy)ethene in the presence of HBF_4_·OEt_2_ or TfOH, Vanallan et al. and Hill used multi-step condensation reaction of salicylaldehyde and acetophenone to prepare 4-phenacylideneflavene derivatives in hot HCl or AcOH [7,8,9]. Although some of these methods are quite useful, there is still further need to develop the simple, efficient, inexpensive, and environment-friendly procedures for the synthesis of 4-phenacylideneflavene.

With an increasing ecological pressure, new green synthetic strategies are of continuously growing interest to synthetize valuable organic compounds due to their environment-friendliness, but this depends on the discovery and usage of mild, low-cost, and high-performance catalysts. Boric acid is a commercially available, environmentally benign, and inexpensive catalyst. Boric acid is a weak solid acid and slightly soluble in water. It forms tetrahydroxyborate in water and serves as a Lewis acid to play catalytic role in various reactions, such as aza-Michael reaction, Biginelli reaction, Mannich reaction, and transamidation of carboxamides [10,11,12,13,14].

**Scheme 1:**
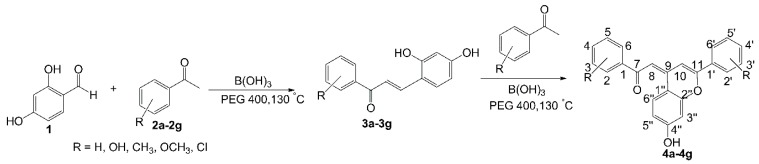
One-pot synthesis of 4-phenacylideneflavene derivatives.

Recently, in the course of our synthetizing 2′,4′-dihydroxychalcone derivatives, our research group accidentally discovered a new way to synthesize 4-phenacylideneflavene derivatives. To the best of our knowledge, no reports for the use of boric acid for condensation of 2,4-dihydroxybenzaldehyde (**1**) and substituted acetophenones (**2a**–**2g**) to form 4-phenacylideneflavenes were reported. Herein, we report a one-pot procedure for the green synthesis of 4-phenacylideneflavenes (**4a**–**4g**) by the condensation of 2,4-dihydroxybenzaldehyde with various substituted acetophenones using boric acid as a green catalyst in polyethylene glycol 400 (Scheme 1). Meanwhile, the products (**4a**–**4g**) were also subjected to cytotoxic tests on liver cancer cell line (HepG2).

## 2. Results and Discussion

### 2.1. Chemistry

The desired seven 4-phenacylideneflavene derivatives (**4a**–**4g**) were prepared using a straightforward one step reaction, as presented in Scheme 1. For the present study, the mixtures of 2,4-dihydroxybenzaldehyde (**1**, 1 mmol) and various substituted acetophenones (**2a**–**2g**, 1 mmol) were stirred in the presence of 0.5 mmol of boric acid in PEG-400 (5 mL) for 6 h at 130 °C. Seven 4-phenacylideneflavene products **4a**–**4g** were isolated by chromatographic purification. The yields of products **4a**–**4g** ranging from 3.06% to 25.28% were determined by HPLC analysis. The yields of products were maybe affected by the molar ratios between 2,4-dihydroxybenzaldehyde and acetophenones and solvent conditions. Taking the product **4e** as example, when the ratio of 2,4-dihydroxybenzaldehyde and 4-methylacetophenone was 1:2, the yield of **4e** had great improvement—up to 58.14%—whereas when the reaction was taken in methanol, no product of **4e** was found. On the other hand, the different substituent groups and substituent positions also affected the yields of products. The results indicated that the conversion to acylideneflavenes preferred electron-rich substituents (R: OH, OMe, etc.) on the aromatic ring than electron-neutral one (R: H). However, electron withdraw groups (R: CN, NO_2_) did not give any desired products under this condition (data not shown). The low yield of **4b** was probably due to the formation of intramolecular hydrogen bond between 2-OH and acetophenone, which inhibited the Aldol reaction and Michael addition afterwards. The structures of the synthesized compounds were characterized by ^1^H-NMR, ^13^C-NMR, and ESI mass spectra. The structures of compounds **4b**–**4d** were finally determined on the base of the 2D-NMR (^1^H-^1^H COSY, HSQC, HMBC, Supplementary materials), whereas the structures of **4a**, **4e**–**4g** were determined on the base of the ^1^H-NMR and ^13^C-NMR and th structure of **4d**. ^1^H-NMR, ^13^C-NMR and mass spectrometric data of synthesized compounds are summarized in Materials and Methods.

**Scheme 2:**
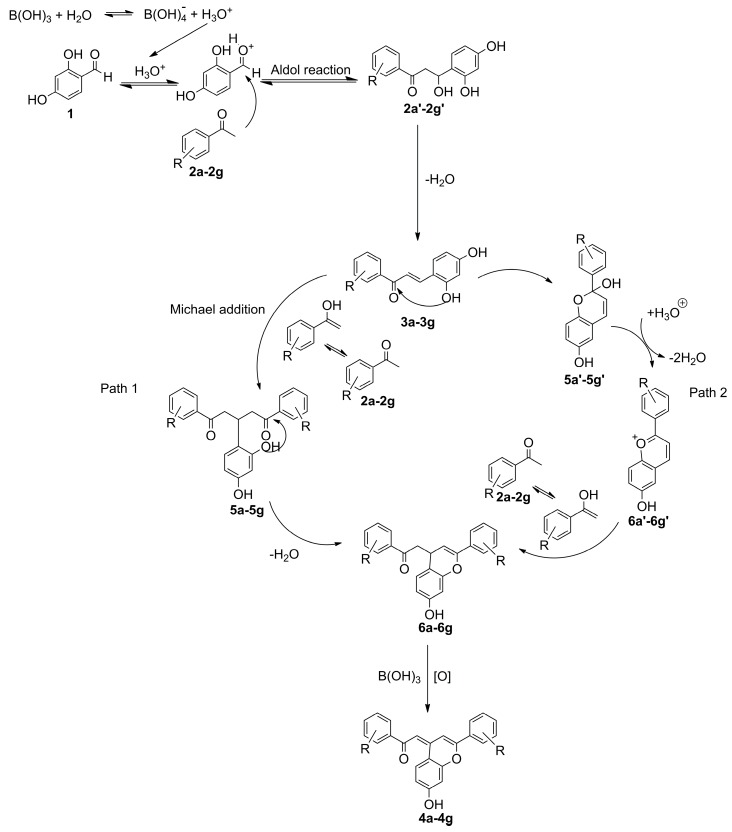
Proposed mechanism for the synthesis of 4-phenacylideneflavene derivatives.

A plausible mechanism for the formation of products **4a**–**4g** was derived and shown in Scheme 2 on the basis of the reported literature [6]. The initial step was Aldol reaction catalyzed by boric acid between 2,4-dihydroxybenzaldehyde (**1**) and substituted acetophenones (**2a**–**2g**) and firstly led to the products of 2′,4′-dihydroxychalcones (**3a**–**3g**). The mechanism in path 1 involved in a Michael addition between substituted acetophenone (**2a**–**2g**) and 2′,4′-dihydroxychalcones (**3a**–**3g**) to offer the products **5a**–**5g**. The products **5a**–**5g** further underwent intramolecular cyclization, followed by dehydration to form adducts (**6a**–**6g**) and then were oxidized to form the product **4a**–**4g**. The other plausible mechanism in path 2 involved in intramolecular cyclization of 2′,4′-dihydroxychalcones (**3a**–**3g**) to form their hemiacetal species **5a′**–**5g′**, which were then converted to more reactive flavylium ions (**6a′**–**6g′**). The flavylium ions (**6a′**–**6g′**) further reacted with substituted acetophenone (**2a**–**2g**) to form adducts (**6a**–**6g**). Finally, adducts were oxidized to form the products **4a**–**4g**, probably through a disproportionation mechanism suggested by VanAllen [8], which also accounted for the low yield of 4-phenacylideneflavene.

### 2.2. Cytotoxic Effects on HepG2 Cell Line

In vitro cytotoxicities of seven 4-phenacylideneflavene derivatives (**4a**–**4g**) were preliminarily evaluated against the selected cancer cell line (HepG2). The results of anticancer activity are expressed as IC_50_ values and are shown in Table 1. As presented in Table 1, among the seven compounds, compound **4e** showed the greatest cytotoxic activity against HepG2 cell. Substitution at the 4 position with methyl group showed significant activity against HepG2, while substitutions with the hydroxyl, methoxyl, and chlorine groups caused substantial decrease in activities, suggesting that the substitution with different groups will cause substantial difference in activities. Thus, more research is worth to carrying out to figure out what group leads to more potential cytotoxicity against HepG2 cell.

## 3. Materials and Methods

### 3.1. General Information

Analytical HPLC was carried out on a Waters 1525 system (Waters, Milford, MA, USA) equipped with a 2487 dual-wavelength detector and the Empower 2 Pro software (Waters, Milford, MA, USA). Alltima C_18_ column (250 × 4.6 mm, 5 μm, Delta Technical Products Co., Des Plaines, IL, USA) was used for analytical HPLC. ^1^H-NMR, ^13^C-NMR, HSQC, and HMBC data were acquired on a Bruker 400 DRX NMR spectrometer (Bruker, Colorado Springs, CO, USA). Molecular weights of compounds were analyzed on Waters Maldi Syapt Q-Tof mass spectrometer (Waters, Milford, MA, USA). Spectrophotometric measurements for the tyrosinase inhibition assay were taken on a UV-5300PC Spectro-photometer (Metash Instrument Co., Ltd., Shanghai, China). 2,4-Dihydroxybenzaldehyde (**1**), acetophenone (**2a**), 2-hydroxyacetophenone (**2b**), 3-hydroxyacetophenone (**2c**), 4-hydroxyacetophenone (**2d**), 4-methylacetophenone (**2e**), 4-methoxyacetophenone (**2f**), and 4-chloroacetophenone (**2g**) were purchased from Shanghai Darui Company (Shanghai, China). Polyethylene glycol 400 (PEG 400), ethanol (EtOH), methanol (MeOH), boric acid, and dichloromethane (CH_2_Cl_2_) were purchased from Sinopharm Chemical Reagent Co., Ltd. (Suzhou, China). Silica gel (200–300 mesh) for column chromatography and TLC plates (HSGF254) were purchased from Yantai Jiangyou Silicone Development Co. (Yantai, China). Dichloromethane (CH_2_Cl_2_), dimethyl sulfoxide (DMSO), 95% ethanol (EtOH), methanol (MeOH), sodium dihydrogen orthophosphate (NaH_2_PO_4_·2H_2_O), formic acid, and anhydrous di-sodium hydrogen phosphate (Na_2_HPO_4_) were purchased from Sinopharm Chemical Reagent Co., Ltd. (Suzhou, China). HPLC grade solvents were purchased from J&K Scientific Ltd. (Beijing, China).

### 3.2. General Procedure for the Synthesis of Compounds ***4a**–**4g***

The mixture of 2,4-dihydroxybenzaldehyde (**1**, 1 mmol), various substituted acetophenone (**2a**–**2g**, 1 mmol), and boric acid (0.5 mmol) were dissolved in PEG-400 and then were stirred to react for 6 h at 130 °C. After the completion of the reaction, the reaction mixture was extracted with ethyl acetate three times. The collected organic layers were combined and concentrated in vacuum, and the residue was subjected to column chromatography on silica gel using CH_2_Cl_2_ (**4a**, **4e**–**4g**) and CH_2_Cl_2_-MeOH (30:1, **4b**–**4d**) as eluent to generate the pure product.

*2-(7-Hydroxy-2-phenyl-chromen-4-ylidene)-1-phenyl-ethanone* (**4a**): Yellow power; m.p. 260 °C; Yield 4.79%; ESI-MS *m*/*z* 339.1 [M − H]^−^; ^1^H-NMR (400 MHz, DMSO-*d*_6_) δ ppm: 10.58 (1H, s, OH-4′′), 8.88 (1H, s, H-10), 8.24 (1H, d, *J* = 9.6 Hz, H-6′′), 8.10 (2H, d, *J* = 7.6 Hz, H-2, 6), 7.95 (2H, d, *J* = 7.2 Hz, H-2′, 6′), 7.58 (2H, d, *J* = Hz, H-3′, 5′), 7.58 (1H, s, H-8), 7.56 (1H, m, H-4), 7.53 (2H, d, *J* = 7.6 Hz, H-3, 5), 7.19 (1H, s, H-4′), 6.90 (2H, overlapped, H-3′′, 5′′); ^13^C-NMR (100 MHz, DMSO-*d*_6_) δ ppm: 188.5 (C=O, C-7), 161.3 (C, C-4′′), 155.1 (C, C-11), 153.9 (C, C-2′′), 142.1 (C, C-1′′), 140.7 (C, C-1′), 132.0 (C, C-1), 131.5 (CH, C-4), 130.7 (CH, C-4′), 129.1 (CH, C-2, 6), 128.37 (CH, C-2′, 6′), 127.6 (CH, C-3, 5), 125.6 (CH, C-6′′), 125.4 (CH, C-3′, 5′), 114.8 (CH, C-10), 111.5 (C, C-9), 103.0 (CH, C-5′′), 101.8 (CH, C-3′′), 101.0 (CH, C-8).

*2-[7-Hydroxy-2-(2-hydroxy-phenyl)-chromen-4-ylidene]-1-(2-hydroxy-phenyl)-ethanone* (**4b**): Red power; m.p. 249 °C; Yield 6.46%; ESI-MS *m*/*z* 371.1 [M − H]^−^; ^1^H-NMR (400 MHz, DMSO-*d*_6_) δ ppm: 13.82 (1H, s, OH-2), 10.65 (2H, s, OH-2′, 4′′), 9.20 (1H, s, H-10), 8.32 (1H, d, *J* = 9.2 Hz, H-6′′), 8.26 (1H, d, *J* = 8.0 Hz, H-6), 7.84 (1H, d, *J* = 7.6 Hz, H-6′), 7.46 (1H, t, *J* = 7.6 Hz, H-4), 7.35 (1H, t, *J* = 8.0 Hz, H-4′), 7.21 (1H, s, H-8), 7.02 (1H, d, *J* = 8.8 Hz, H-3′), 6.98 (1H, d, *J* = 7.6 Hz, H-3), 6.93 (1H, overlapped, H-5′), 6.92 (1H, overlapped, H-5), 6.90 (1H, overlapped, H-3′′), 6.88 (1H, overlapped, H-5′′); ^13^C-NMR (100 MHz, DMSO-*d*_6_) δ ppm: 192.3 (C=O, C-7), 162.3 (C, C-2), 161.6 (C, C-4′′), 156.2 (C, C-2′), 154.4 (C, C-11), 154.3 (C, C-2′′), 145.0 (C, C-1′′), 134.6 (CH, C-4), 131.7 (CH, C-4′), 129.4 (CH, C-6), 128.3 (CH, C-6′), 126.0 (CH, C-6′′), 121.8 (C, C-1), 119.3 (CH, C-3), 118.6 (C, C-1′), 118.4 (CH, C-5′), 117.7 (CH, C-5), 116.8 (CH, C-3′), 115.0 (CH, C-5′′), 111.6 (C, C-9), 106.6 (CH, C-10), 102.8 (CH, C-3′′), 98.6 (CH, C-8).

*2-[7-Hydroxy-2-(3-hydroxy-phenyl)-chromen-4-ylidene]-1-(3-hydroxy-phenyl)-ethanone* (**4c**): Red power; m.p. 310 °C; Yield 19.27%; ESI-MS *m*/*z* 371.1 [M − H]^−^; ^1^H-NMR (400 MHz, DMSO-*d*_6_) δ ppm: 10.56 (1H, s, OH-4′′), 9.82 (1H, s, OH-3), 9.62 (1H, s, OH-3′), 8.79 (1H, s, H-10), 8.18 (1H, d, *J* = 9.2 Hz, H-6′′), 7.55 (1H, d, *J* = 8.0 Hz, H-6), 7.41 (1H, H-4), 7.38 (2H, overlapped, H-5′, 6′), 7.35 (1H, H-4′), 7.31 (1H, t, *J* = 8.0 Hz, H-5), 7.10 (1H, s, H-8), 6.97 (1H, H-2), 6.95 (1H, H-2′), 6.88 (1H, dd, *J* = 8.8, 2.4 Hz, H-5′′), 6.85 (1H, d, *J* = 2.4 Hz, H-3′′); ^13^C-NMR (100 MHz, DMSO-*d*_6_) δ ppm: 188.5 (C=O, C-7), 161.2 (C, C-4′′), 157.8 (C, C-3′), 157.4 (C, C-3), 155.0 (C, C-11), 153.8 (C, C-2′′), 142.2 (C, C-1), 141.9 (C, C-1′′), 133.2 (C, C-1′), 130.2 (CH, C-5′), 129.3 (CH, C-5), 125.5 (CH, C-6′′), 118.6 (CH, C-6), 118.4 (CH, C-2), 117.9 (CH, C-2′), 116.2 (CH, C-6′), 114.8 (CH, C-5′′), 114.0 (CH, C-4), 111.9 (CH, C-4′), 111.5 (C, C-9), 102.9 (CH, C-3′′), 101.7 (CH, C-10), 101.0 (CH, C-8).

*2-[7-Hydroxy-2-(4-hydroxy-phenyl)-chromen-4-ylidene]-1-(4-hydroxy-phenyl)-ethanone* (**4d**): Red power; m.p. 197 °C; Yield 3.06%; ESI-MS *m*/*z* 371.2 [M − H]^−^; ^1^H-NMR (400 MHz, DMSO-*d*_6_) δ ppm: 10.15 (3H,br s, OH-4, 4′, 4′′), 8.74 (1H, s, H-10), 8.17 (1H, d, *J* = 8.8 Hz, H-6′′), 8.00 (2H, d, *J* = 8.8 Hz, H-2, 6), 7.79 (2H, d, *J* = 8.4 Hz, H-2′, 6′), 7.09 (1H, s, H-8), 6.96 (2H, d, *J* = 8.8 Hz, H-3′, 5′), 6.86 (2H, d, *J* = 8.8 Hz, H-3, 5), 6.85 (2H, overlapped, H-3′′, 5′′); ^13^C-NMR (100 MHz, DMSO-*d*_6_) δ ppm: 187.2 (C=O, C-7), 160.9 (C, C-4′′), 160.7 (C, C-4), 159.9 (C, C-4′), 155.0 (C, C-11), 153.7 (C, C-2′′), 141.4 (C, C-1′′), 132.2 (C, C-1), 129.9 (CH, C-2, 6), 127.2, 127.1 (CH, C-2′, 6′), 125.4 (CH, C-6′′), 122.7 (C, C-1′), 116.0, 115.8 (CH, C-3′, 5′), 115.0, 114.8 (CH, C-3, 5), 111.7 (C, C-9), 103.1, 102.8 (CH, C-3′′, 5′′), 99.8 (CH, C-8, 10).

*2-(7-Hydroxy-2-p-tolyl-chromen-4-ylidene)-1-p-tolyl-ethanone* (**4e**): Yellow power; m.p. 258 °C; Yield 25.28%; ESI-MS *m*/*z* 367.2 [M − H]^−^; ^1^H-NMR (400 MHz, DMSO-*d*_6_) δ ppm: 10.52 (1H, s, OH-4′′), 8.84 (1H, s, H-10), 8.20 (1H, d, *J* = 8.8 Hz, H-6′′), 8.00 (2H, d, *J* = 8.0 Hz, H-2, 6), 7.82 (2H, d, *J* = 8.4 Hz, H-2′, 6′), 7.37 (2H, d, *J* = 8.4 Hz, H-3′, 5′), 7.31 (2H, d, *J* = 8.0 Hz, H-3, 5), 7.15 (1H, s, H-8), 6.88 (1H, dd, *J* = 8.0, 2.4 Hz, H-5′′), 6.87 (1H, d, *J* = 2.4 Hz, H-3′′); ^13^C-NMR (100 MHz, DMSO-*d*_6_) δ ppm: 188.7 (C=O, C-7), 161.2 (C, C-4′′), 155.0 (C, C-11), 153.8 (C, C-2′′), 141.9 (C, C-1′′), 141.6 (C, C-4), 140.7 (C, C-4′), 138.1 (C, C-1), 129.7 (CH, C-2, 6), 129.2 (C, C-1′), 129.0 (CH, C-2′, 6′), 127.7 (CH, C-3, 5), 125.6 (CH, C-6′′), 125.3 (CH, C-3′, 5′), 114.7 (CH, C-10), 111.6 (C, C-9), 103.0 (CH, CH-5′′), 101.2 (CH, C-3′′), 100.6 (CH, C-8), 21.0 (CH_3_-4, 4′).

*2-[7-Hydroxy-2-(4-methoxy-phenyl)-chromen-4-ylidene]-1-(4-methoxy-phenyl)-ethanone* (**4f**): Yellow power; m.p. 260 °C; Yield 19.37%; ESI-MS *m*/*z* 399.2 [M − H]^−^; ^1^H-NMR (400 MHz, DMSO-*d*_6_) δ ppm: 8.78 (1H, s, H-10), 8.20 (1H, d, *J* = 9.6 Hz, H-6′′), 8.09 (2H, d, *J* = 8.8 Hz, H-2, 6), 7.88 (2H, d, *J* = 8.4 Hz, H-2′, 6′), 7.13 (2H, d, *J* = 8.8 Hz, H-3′, 5′), 7.121 (1H, s, H-8), 7.03 (2H, d, *J* = 8.8 Hz, H-3, 5), 6.87 (1H, overlapped, H-3′′), 6.86 (1H, overlapped, H-5′′); ^13^C-NMR (100 MHz, DMSO-*d*_6_) δ ppm: 187.3 (C=O, C-7), 162.0 (C, C-4′′), 161.3 (C, C-4), 161.1 (C, C-4′), 154.9 (C, C-11), 153.8 (C, C-2′′), 141.7 (C, C-1′′), 133.6 (C, C-1), 129.8 (CH, C-2, 6), 127.2 (CH, C-2′, 6′), 125.5 (CH, C-6′′), 124.3 (C, C-1′), 114.6 (CH, C-3′, 5′), 114.6 (CH, C-10), 113.6 (CH, C-3, 5), 111.7 (C, C-9), 103.0 (CH, CH-5′′), 100.4 (CH, C-3′′), 100.2 (CH, C-8), 55.5 (CH_3_O-4), 55.4 (CH_3_O-4′).

*1-(4-Chloro-phenyl)-2-[2-(4-chloro-phenyl)-7-hydroxy-chromen-4-ylidene]-ethanone* (**4g**): Yellow power; m.p. 298 °C; Yield 20.45%; ESI-MS *m*/*z* 407.1 [M − H]^−^; ^1^H-NMR (400 MHz, DMSO-*d*_6_) δ ppm: 8.79 (1H, s, H-10), 8.16 (1H, d, *J* = 8.8 Hz, H-6′′), 8.08 (2H, d, *J* = 8.4 Hz, H-2, 6), 7.92 (2H, d, *J* = 8.4 Hz, H-2′, 6′), 7.60 (2H, d, *J* = 8.4 Hz, H-3′, 5′), 7.53 (2H, d, *J* = 8.4 Hz, H-3, 5), 7.12 (1H, s, H-8), 6.89 (1H, dd, *J* = 8.8, 2.0 Hz, H-5′′), 6.86 (1H, d, *J* = 2.4 Hz, H-3′′); ^13^C-NMR (100 MHz, DMSO-*d*_6_) δ ppm: 187.0 (C=O, C-7), 161.3 (C, C-4′′), 153.6 (C, C-11), 153.7 (C, C-2′′), 142.2 (C, C-1′′), 139.2 (C, C-1), 136.3 (C, C-4), 135.3 (C, C-4′), 130.7 (C, C-1′), 129.2 (CH, C-2, 6), 128.2 (CH, C-2′, 6′), 127.0 (CH, C-3, 5), 125.3 (CH, C-6′′), 114.8 (CH, C-10), 111.3 (C, C-9), 102.9 (CH, CH-5′′), 102.1 (CH, C-3′′), 100.8 (CH, C-8).

### 3.3. HPLC Analysis of the Products

The yields of products were determined by HPLC analysis on the crude reaction mixture after having made a calibration curve with pure compounds isolated by chromatography. The analytical HPLC system consisted of a Shimadzu LC-20AT series pumping system, an SIL-20A automatic injector, an SPD-M20A UV-visible detector, and Class-Vp chromatography data station software. All samples were analyzed by HPLC using a reverse-phase GraceSmart column (4.6 μm, 2.1 × 250 mm, Ryss Tech Ltd., Shanghai, China) at 30 °C with a flow rate of 1.0 mL/min. The mobile phases consisted of solvent A (0.1% formic acid in water, *v*/*v*) and solvent B (methanol). The gradient elution was as follows: initially, 20% B; 0–10 min, 50% B; 10–30 min, 80% B; 30–32 min, 100% B; 32–35 min, 100% B; 35–40 min, 20% B; 40–50 min, 20% B. Flow rate was set at 1.0 mL/min. The sample injection volume was 10 μL. The UV detector was set at 369 nm.

### 3.4. Biological Evaluation

#### 3.4.1. Cell Culture

HepG2 cell was cultured in Dulbecco’s Modified Eagle Medium (high glucose) and RPMI-1640, respectively, with 10% fetal bovine serum under standard culture conditions. When the cells grew to about 80% confluence, they were sub-cultured or treated with products.

#### 3.4.2. Growth Inhibition Study

The proliferation of cells was assessed by using the 3-(4,5-dimethylthiazol-2-yl)-2,5-diphenyltetrazolium bromide (MTT) assay. Cells were plated on 96-well microplates in 100 μL (1 × 10^4^/well). After 12 h, the cells were treated with the medium (without FBS) containing compounds (12.5, 25, and 50 μM) for 24 h. At the end of experiments, 10 μL of 5 μg/mL MTT was directly added to each well. Cells were then incubated at 37 °C for 4 h. After removing medium, formazan was solubilized by 100 μL of DMSO and measured at 560 nm. All experiments were repeated three times.

## 4. Conclusions

In conclusion, a simple one pot synthesis of 4-phenacylideneflavene derivatives from the reaction between 2,4-dihydroxybenzaldehyde and substituted acetophenones had been developed in the presence of boric acid as catalyst in PEG 400. The method provided an inexpensive, safe, simple, and eco-friendly way to synthetize 4-phenacylideneflavene derivatives. On the other hand, the synthesized 4-phenacylideneflavene products were found to have potential inhibitory activities against HepG2 cell line. To the best of our knowledge, this is the first report for the use of boric acid for condensation of 2,4-dihydroxybenzaldehyde and substituted acetophenones to form 4-phenacylideneflavenes and preliminarily evaluated their anticancer activities against HepG2 cell line, which will contribute to the development of green strategy for synthesizing various biologically flavene products.

## Figures and Tables

**Table 1:** Inhibitory effects of the products against HepG2 cell line

Products	R	HepG2 (IC_50_ µM)
**4a**	H	20–50
**4b**	2-OH	20–50
**4c**	3-OH	>50
**4d**	4-OH	>50
**4e**	4-CH_3_	≈12.5 ^a^
**4f**	4-OCH_3_	20–50
**4g**	4-Cl	>50

^a^ Values are means ± SD (*n* = 3).

## Supplementary Materials

The 1D- (^1^H- and ^13^C-NMR), 2D-NMR (^1^H-^1^H COSY, HSQC and HMBC), ESI-MS spectra of compounds are available in the supplementary materials.
