# Supplementary material for: A New Approach to Synthesize of 4-Phenacylideneflavene Derivatives and to Evaluate Their Cytotoxic Effects on HepG2 Cell Line
Source: Molecules. 2017 Aug 9;22(8):1296. doi: 10.3390/molecules22081296 (PMC6152225; doi:10.3390/molecules22081296)
Supplement: Supplementary file 1 [file molecules-22-01296-s001.pdf]

Supplementary materials for

A new approach to synthesis of 4-phenacylideneflavene derivatives and their potential cytotoxic effects on HepG2 cell line

Hongbin Chen[1], Yang Xu[2], Yi-Nan Zhang[3], Zong-Ping Zheng[1,*]

[1]Fujian Province Key Laboratory for the Development of Bioactive material from marine alge, College of Oceanology and Food Science, Quanzhou Normal University, Quanzhou 362000, Fujian Province, Republic of China

[2]School of Pharmaceutical Sciences, Xiamen University, Xiamen, Fujian, People's Republic of China

[3]College of pharmacy, University of Kentucky, Lexington, Kentucky 40536, United States

*Corresponding author. Tel./Fax: +86 595 22919563.

E-mail address: zzpsea@qztc.edu.cn (Z.P. Zheng).

Figure S1. ESI-MS of 2-(7-hydroxy-2-phenyl-chromen-4-ylidene)-1-phenyl-ethanone (**4a**)
Figure S2. ^{1}H NMR of 2-(7-hydroxy-2-phenyl-chromen-4-ylidene)-1-phenyl-ethanone (**4a**)
Figure S3. ^{13}C NMR of 2-(7-hydroxy-2-phenyl-chromen-4-ylidene)-1-phenyl-ethanone (**4a**)
Figure S4. ESI-MS of 2-[7-hydroxy-2-(2-hydroxy-phenyl)-chromen-4-ylidene]-1-(2-hydroxy-phenyl)-ethanone (**4b**)
Figure S5. ^{1}H NMR of of 2-[7-hydroxy-2-(2-hydroxy-phenyl)-chromen-4-ylidene]-1-(2-hydroxy-phenyl)-ethanone (**4b**)
Figure S5. ^{13}C NMR of of 2-[7-hydroxy-2-(2-hydroxy-phenyl)-chromen-4-ylidene]-1-(2-hydroxy-phenyl)-ethanone (**4b**)
Figure S6. ^{1}H-^{1}H COSY of 2-[7-hydroxy-2-(2-hydroxy-phenyl)-chromen-4-ylidene]-1-(2-hydroxy-phenyl)-ethanone (**4b**)
Figure S7. HSQC of 2-[7-hydroxy-2-(2-hydroxy-phenyl)-chromen-4-ylidene]-1-(2-hydroxy-phenyl)-ethanone (**4b**)
Figure S8. HMBC of 2-[7-hydroxy-2-(2-hydroxy-phenyl)-chromen-4-ylidene]-1-(2-hydroxy-phenyl)-ethanone (**4b**)
Figure S9. ESI-MS of 2-[7-hydroxy-2-(3-hydroxy-phenyl)-chromen-4-ylidene]-1- (3-hydroxy-phenyl)-ethanone (**4c**)
Figure S10. ^{1}H NMR of 2-[7-hydroxy-2-(3-hydroxy-phenyl)-chromen-4-ylidene]-1- (3-hydroxy-phenyl)-ethanone (**4c**)
Figure S11. ^{13}C NMR of 2-[7-hydroxy-2-(3-hydroxy-phenyl)-chromen-4-ylidene]-1-(3-hydroxy-phenyl)-ethanone (**4c**)
Figure S12. HSQC of 2-[7-hydroxy-2-(3-hydroxy-phenyl)-chromen-4-ylidene]-1- (3-hydroxy-phenyl)-ethanone (**4c**)
Figure S13. HMBC of 2-[7-hydroxy-2-(3-hydroxy-phenyl)-chromen-4-ylidene]-1- (3-hydroxy-phenyl)-ethanone (**4c**)
Figure S14. ESI-MS of 2-[7-hydroxy-2-(4-hydroxy-phenyl)-chromen-4-ylidene]-1-(4-hydroxy-phenyl)-ethanone (**4d**)
Figure S15. ^{1}H NMR of 2-[7-hydroxy-2-(4-hydroxy-phenyl)-chromen-4-ylidene]-1- (4-hydroxy-phenyl)-ethanone (**4d**)
Figure S16. ^{13}C NMR of 2-[7-hydroxy-2-(4-hydroxy-phenyl)-chromen-4-ylidene]-1-(4-hydroxy-phenyl)-ethanone (**4d**)
Figure S17. ^{1}H-^{1}H COSY of 2-[7-hydroxy-2-(4-hydroxy-phenyl)-chromen-4-ylidene]-1-(4-hydroxy-phenyl)-ethanone (**4d**)
Figure S18. HSQC of 2-[7-hydroxy-2-(4-hydroxy-phenyl)-chromen-4-ylidene]-1- (4-hydroxy-phenyl)-ethanone (**4d**)
Figure S19. HMBC of 2-[7-hydroxy-2-(4-hydroxy-phenyl)-chromen-4-ylidene]-1- (4-hydroxy-phenyl)-ethanone (**4d**)
Figure S20. ESI-MS of 2-(7-Hydroxy-2-p-tolyl-chromen-4-ylidene)-1-p-tolyl-ethanone (**4e**)
Figure S21. ^{1}H NMR of 2-(7-Hydroxy-2-p-tolyl-chromen-4-ylidene)-1-p-tolyl-ethanone (**4e**)
Figure S22. ^{13}C NMR of 2-(7-Hydroxy-2-p-tolyl-chromen-4-ylidene)-1-p-tolyl-ethanone (**4e**)
Figure S23. ESI-MS of 2-[7-hydroxy-2-(4-methoxy-phenyl)-chromen-4-ylidene]-1-(4-methoxy-

phenyl)-ethanone (**4f**)

Figure S24. ^{1}H NMR of 2-[7-hydroxy-2-(4-methoxy-phenyl)-chromen-4-ylidene]-1-(4-methoxy-phenyl)-ethanone (**4f**)

Figure S25. ^{13}C NMR of 2-[7-hydroxy-2-(4-methoxy-phenyl)-chromen-4-ylidene]-1-(4-methoxy-phenyl)-ethanone (**4f**)

Figure S26. ESI-MS of 1-(4-Chloro-phenyl)-2-[2-(4-chloro-phenyl)-7-hydroxy-chromen-4-ylidene]-ethanone (**4g**)

Figure S27. ^{1}H NMR of 1-(4-Chloro-phenyl)-2-[2-(4-chloro-phenyl)-7-hydroxy-chromen-4-ylidene]-ethanone (**4g**)

Figure S28. ^{13}C NMR of 1-(4-Chloro-phenyl)-2-[2-(4-chloro-phenyl)-7-hydroxy-chromen-4-ylidene]-ethanone (**4g**)

Figure S29. The structures of compounds **4a-4g**

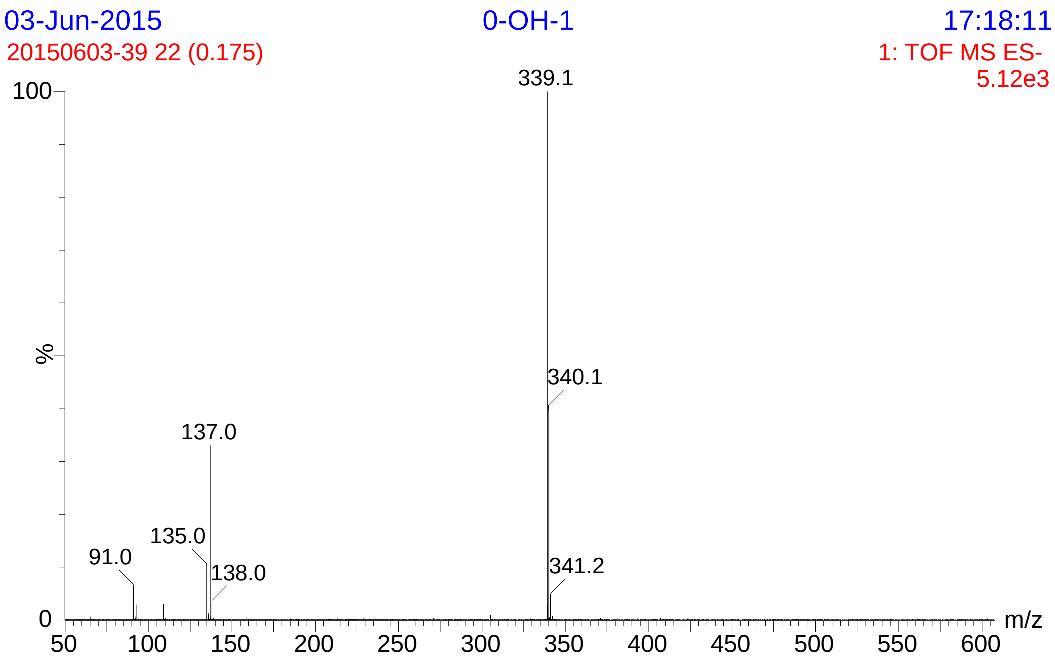

Figure S1. ESI-MS of 2-(7-hydroxy-2-phenyl-chromen-4-ylidene)-1-phenyl-ethanone (**4a**)

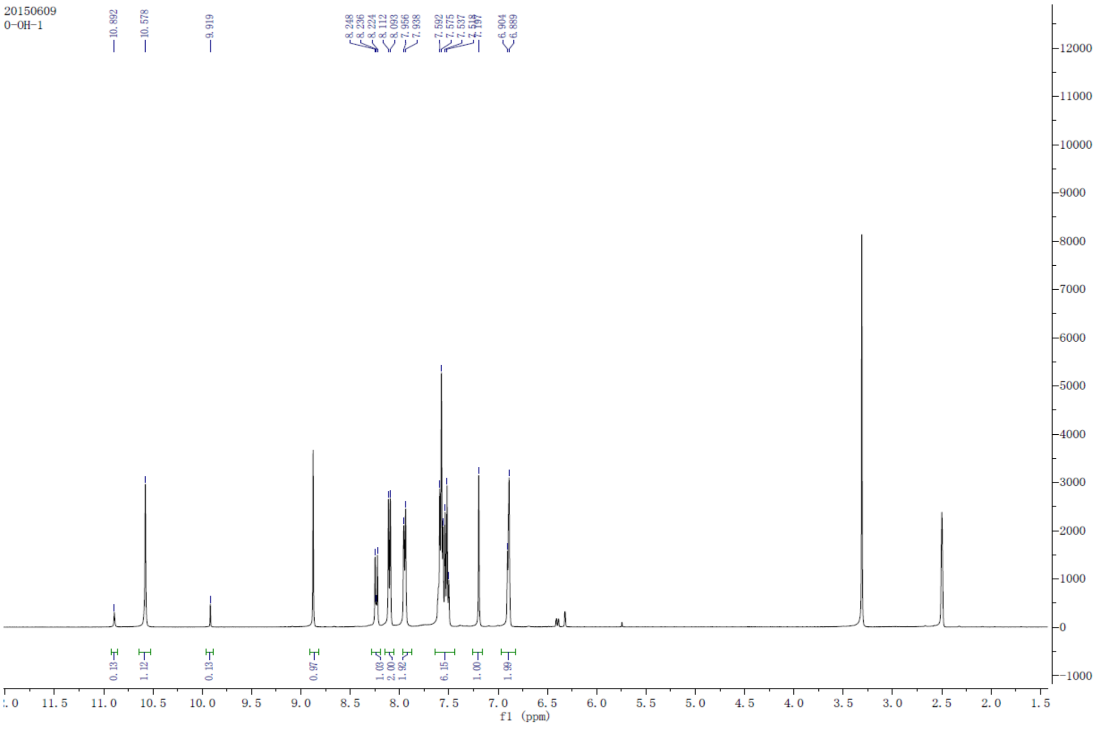

Figure S2. ^{1}H NMR of 2-(7-hydroxy-2-phenyl-chromen-4-ylidene)-1-phenyl-ethanone (**4a**)

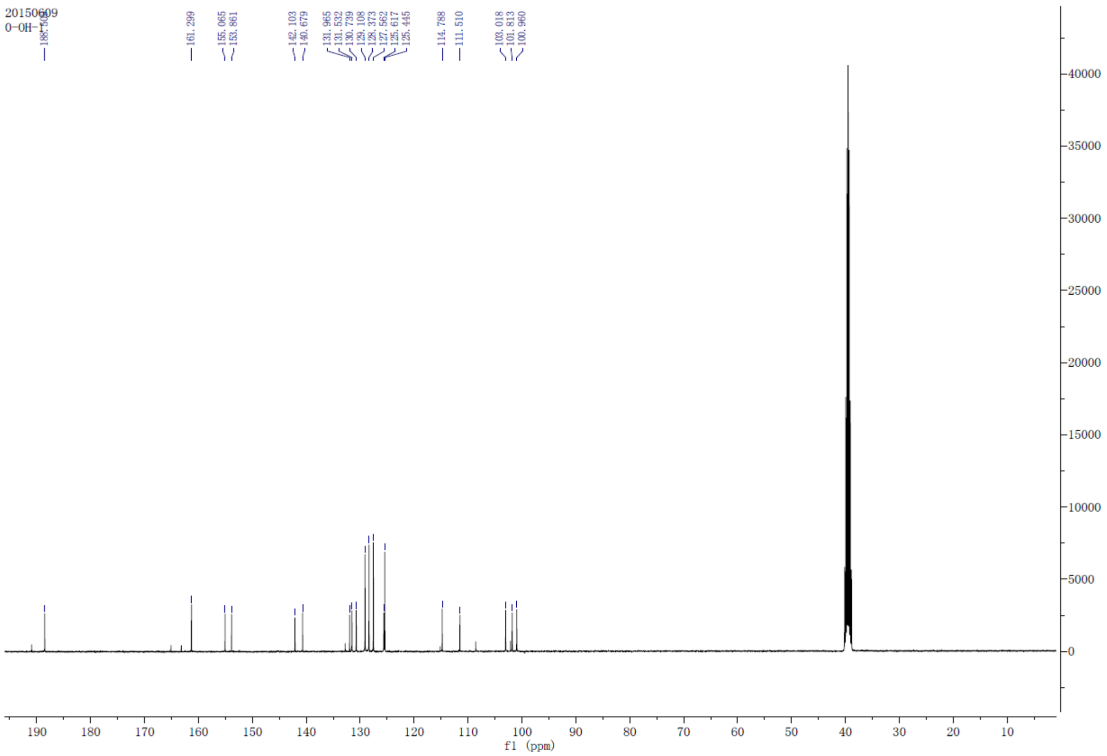

Figure S3. ^{13}C NMR of 2-(7-hydroxy-2-phenyl-chromen-4-ylidene)-1-phenyl-ethanone (**4a**)

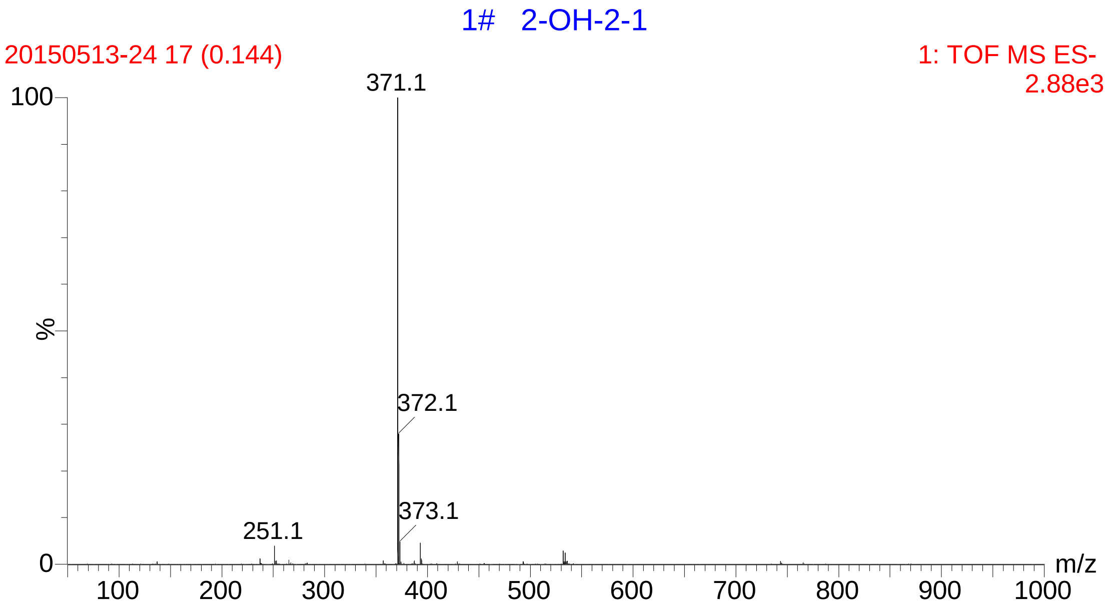

Figure S4. ESI-MS of 2-[7-hydroxy-2-(2-hydroxy-phenyl)-chromen-4-ylidene]-1-(2-hydroxy-phenyl)-ethanone (**4b**)

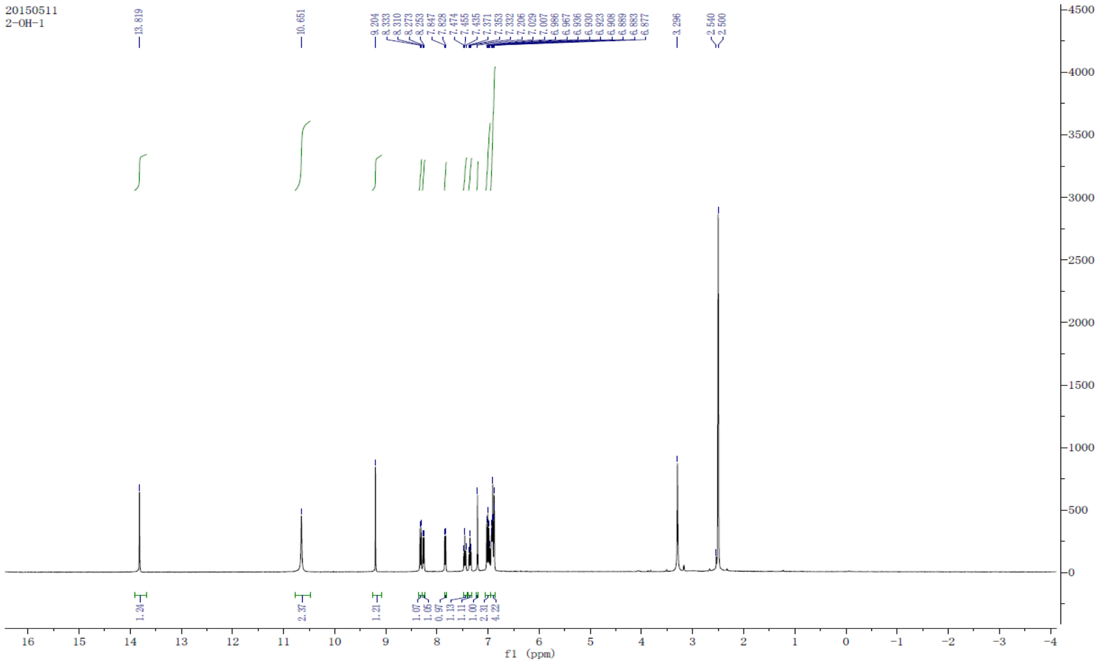

Figure S5. ^{1}H NMR of of 2-[7-hydroxy-2-(2-hydroxy-phenyl)-chromen-4-ylidene]-1-(2-hydroxy- phenyl)-ethanone (**4b**)

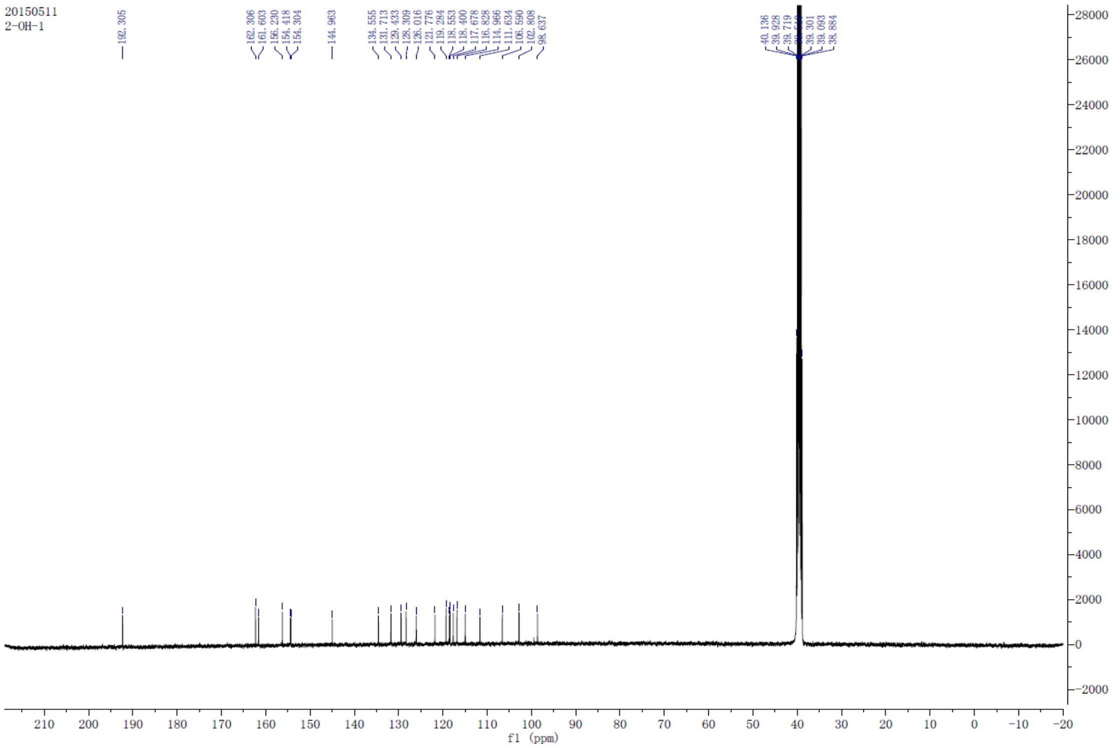

Figure S5. ^{13}C NMR of of 2-[7-hydroxy-2-(2-hydroxy-phenyl)-chromen-4-ylidene]-1-(2-hydroxy-phenyl)-ethanone (**4b**)

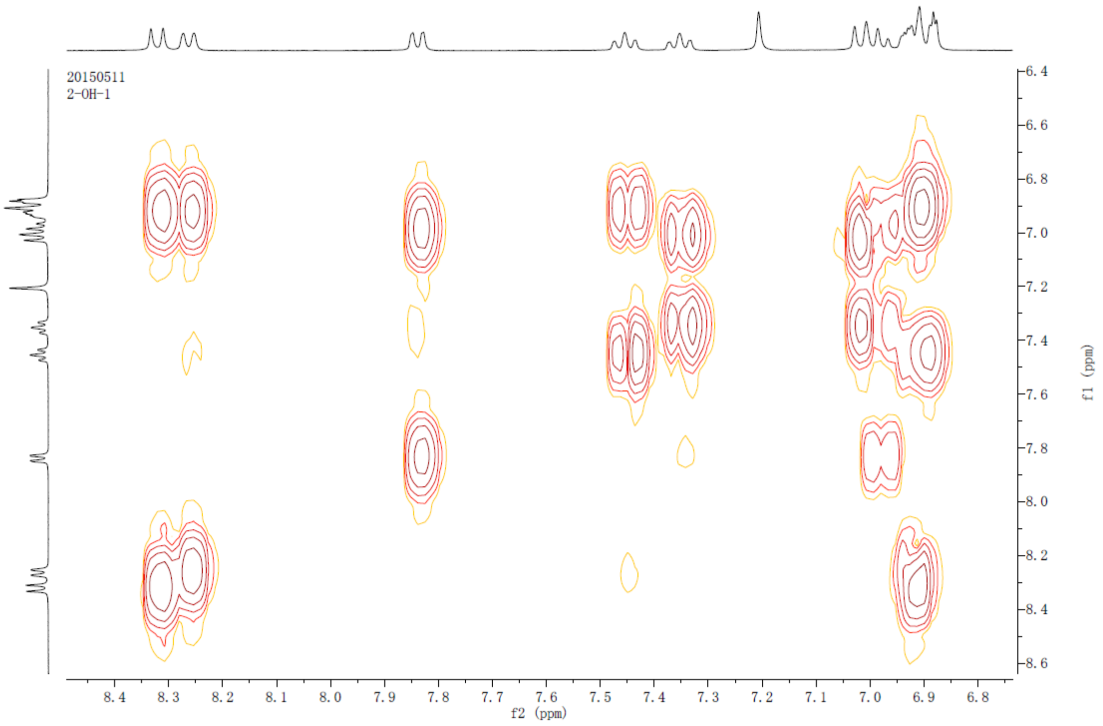

Figure S6. ^{1}H-^{1}H COSY of 2-[7-hydroxy-2-(2-hydroxy-phenyl)-chromen-4-ylidene]-1-(2-hydroxy-phenyl)-ethanone (**4b**)

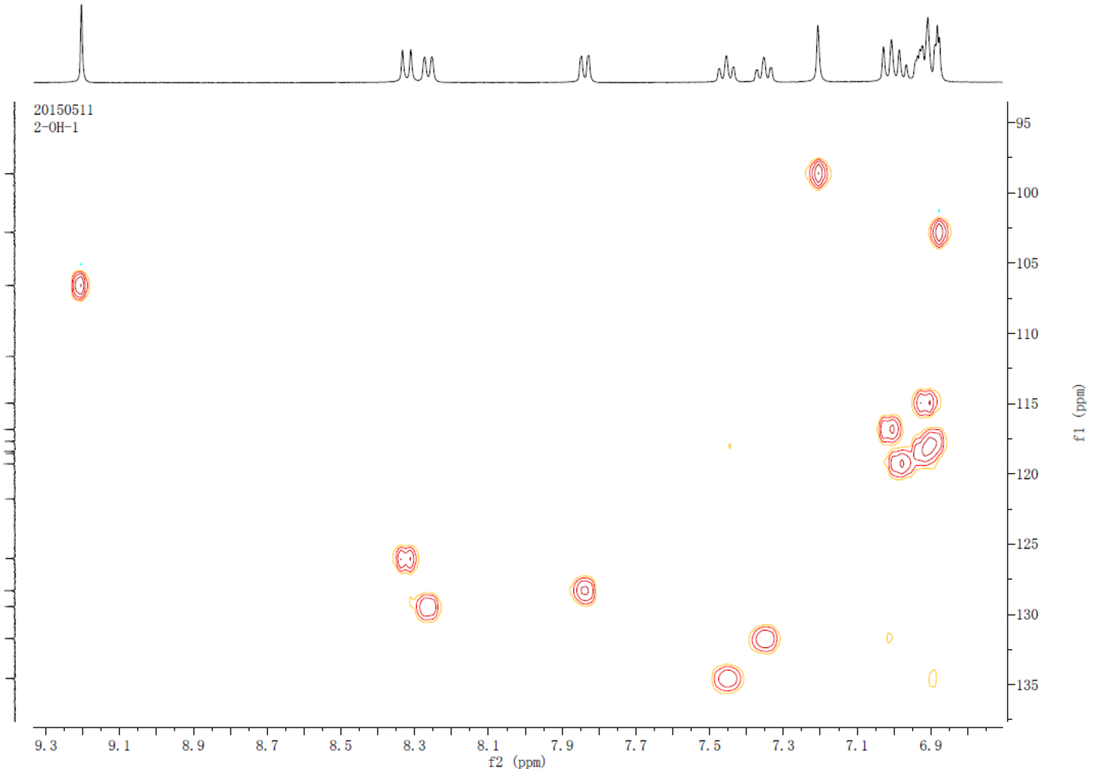

Figure S7. HSQC of 2-[7-hydroxy-2-(2-hydroxy-phenyl)-chromen-4-ylidene]-1-(2-hydroxy-phenyl)-ethanone (**4b**)

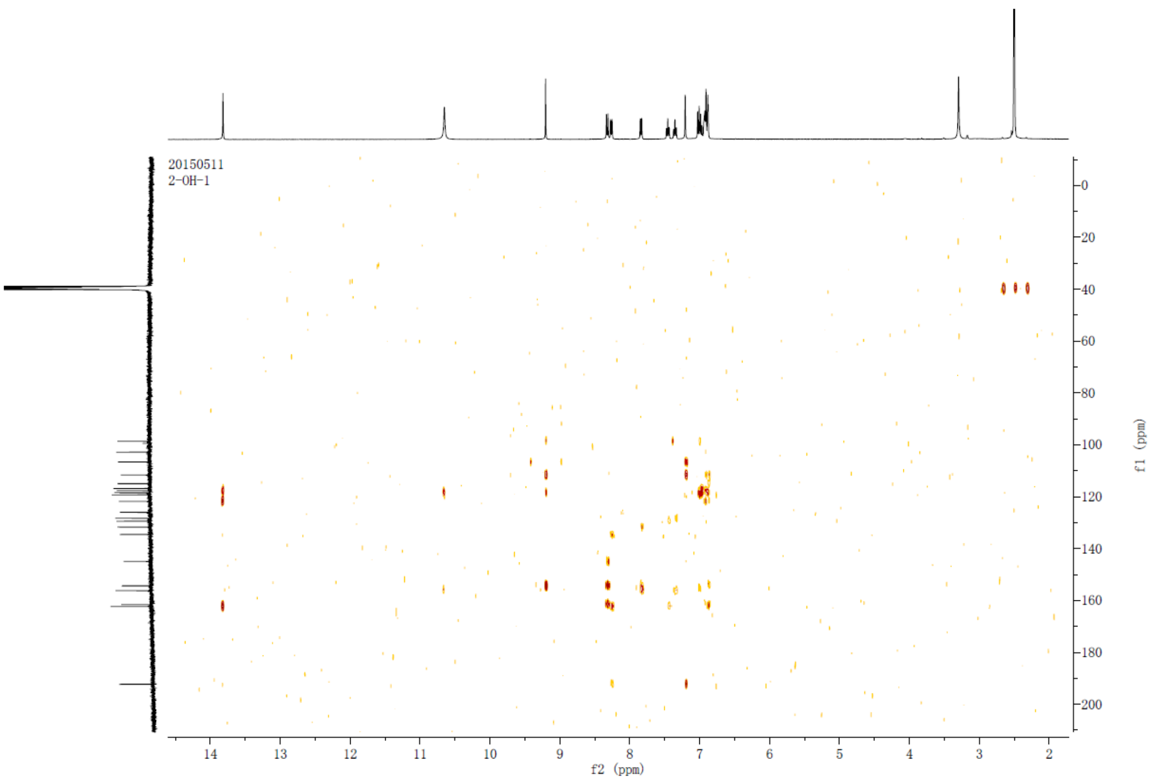

Figure S8. HMBC of 2-[7-hydroxy-2-(2-hydroxy-phenyl)-chromen-4-ylidene]-1-(2-hydroxy-phenyl)-ethanone (**4b**)

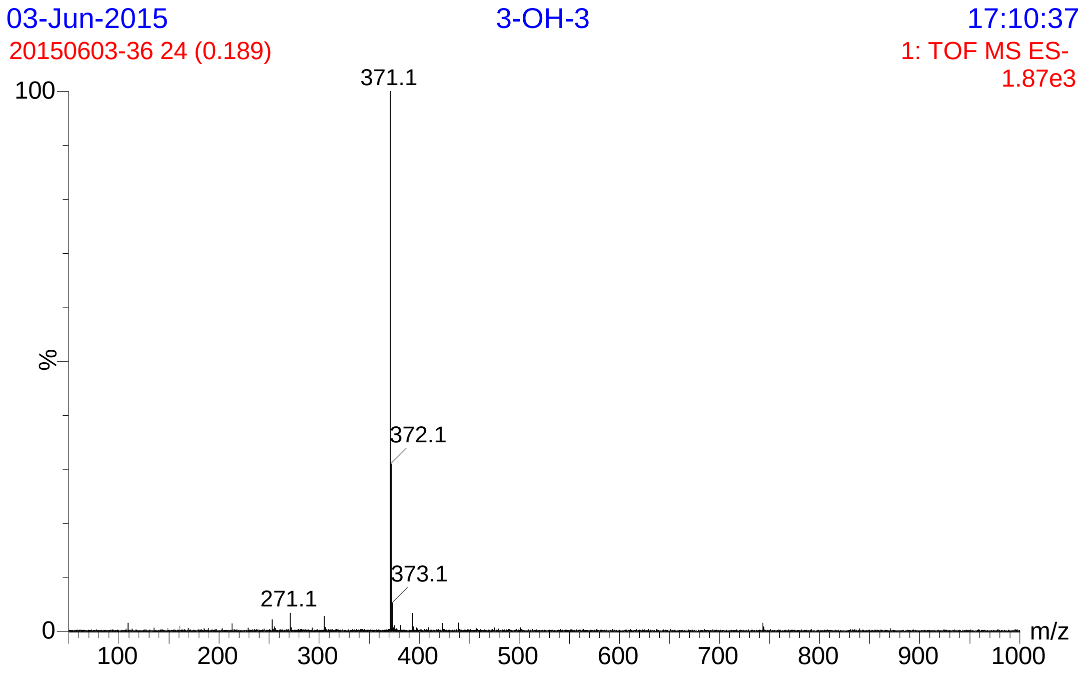

Figure S9. ESI-MS of 2-[7-hydroxy-2-(3-hydroxy-phenyl)-chromen-4-ylidene]-1-(3-hydroxy-phenyl)-ethanone (**4c**)

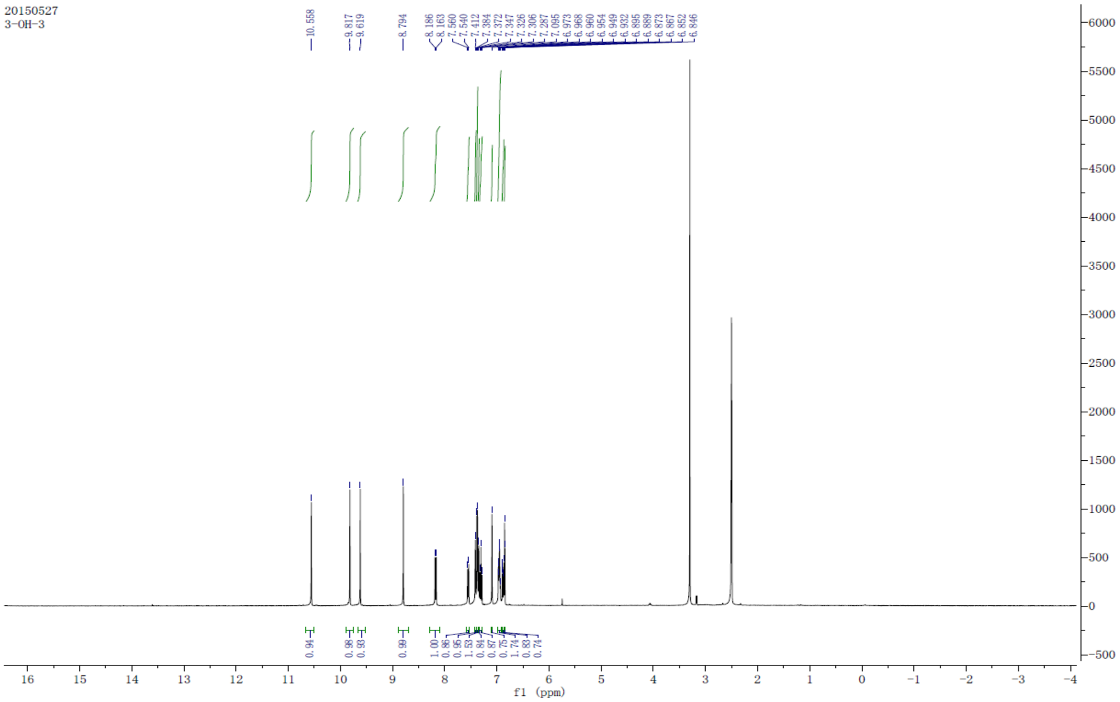

Figure S10. ^{1}H NMR of 2-[7-hydroxy-2-(3-hydroxy-phenyl)-chromen-4-ylidene]-1-(3-hydroxy-phenyl)-ethanone (**4c**)

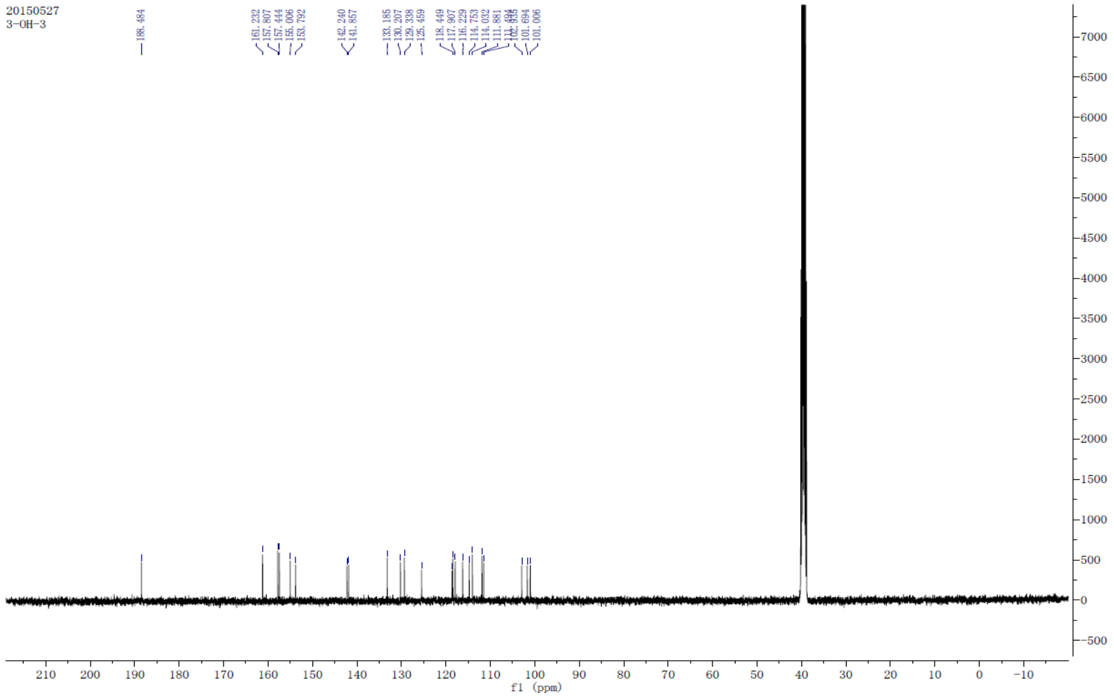

Figure S11. ^{13}C NMR of 2-[7-hydroxy-2-(3-hydroxy-phenyl)-chromen-4-ylidene]-1-(3-hydroxy-phenyl)-ethanone (**4c**)

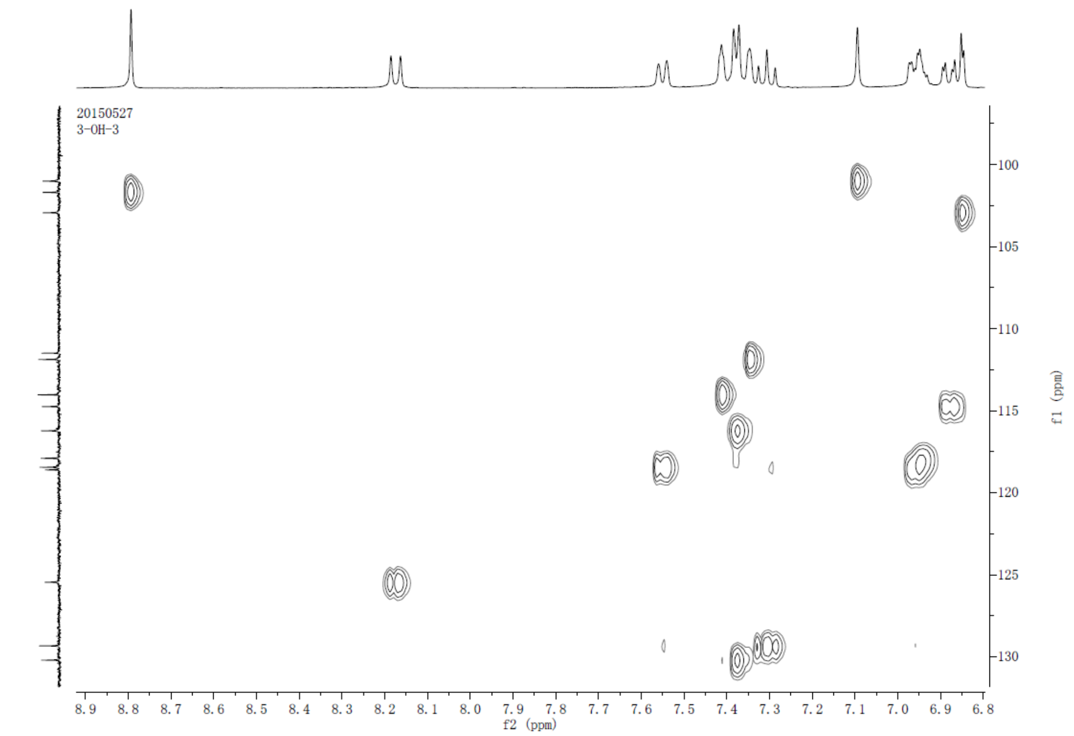

Figure S12. HSQC of 2-[7-hydroxy-2-(3-hydroxy-phenyl)-chromen-4-ylidene]-1-(3-hydroxy-phenyl)-ethanone (**4c**)

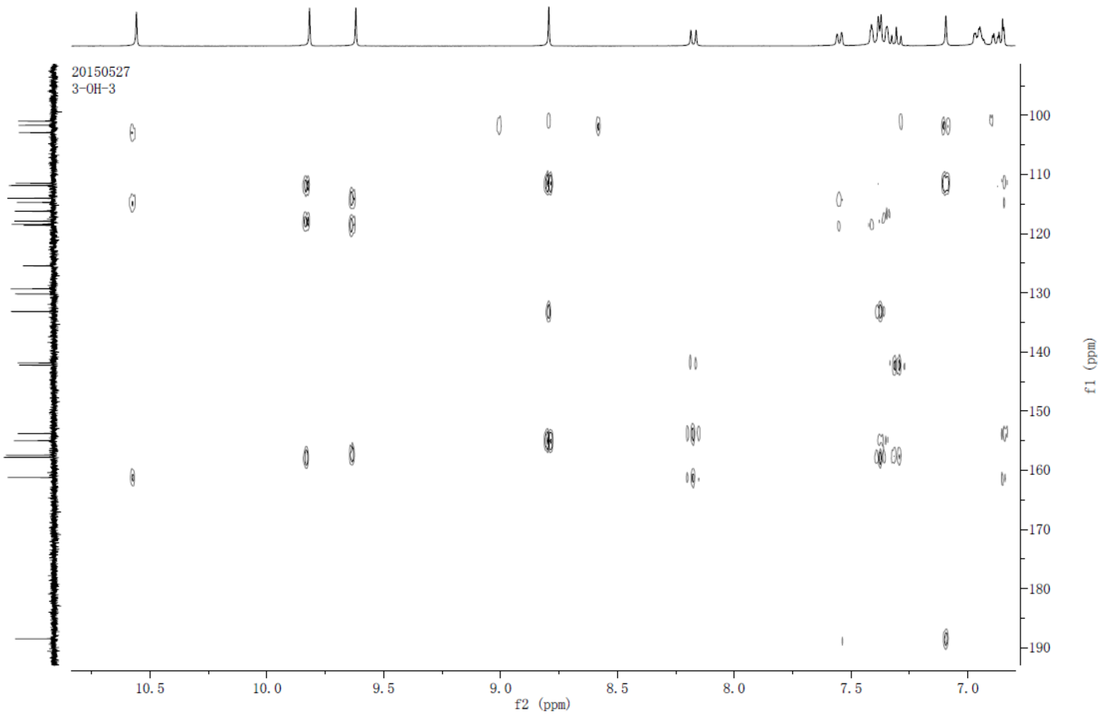

Figure S13. HMBC of 2-[7-hydroxy-2-(3-hydroxy-phenyl)-chromen-4-ylidene]-1-(3-hydroxy-phenyl)-ethanone (**4c**)

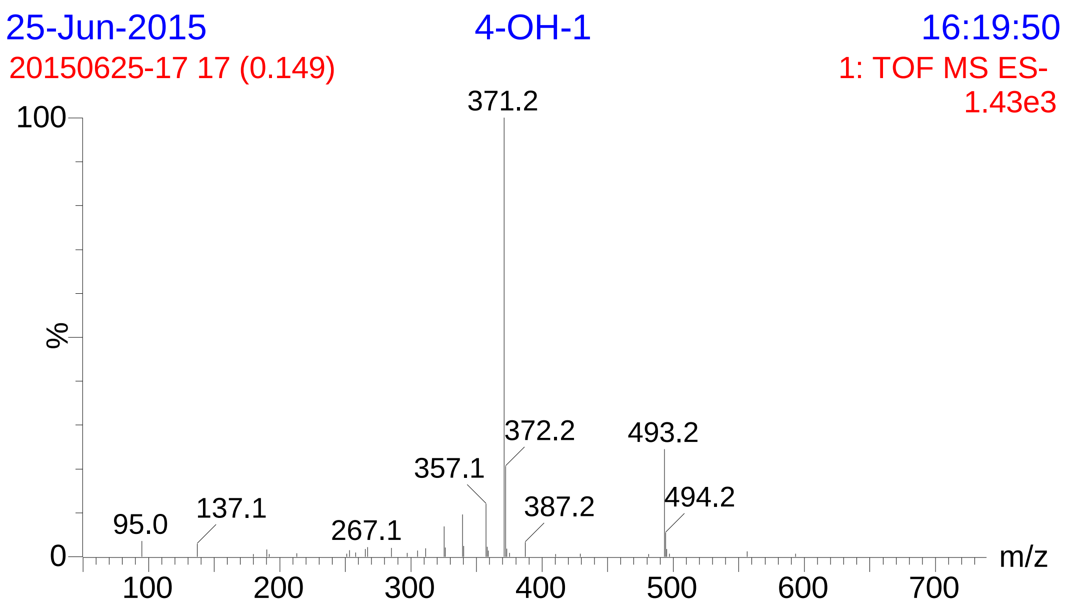

Figure S14. ESI-MS of 2-[7-hydroxy-2-(4-hydroxy-phenyl)-chromen-4-ylidene]-1-(4-hydroxy-phenyl)-ethanone (**4d**)

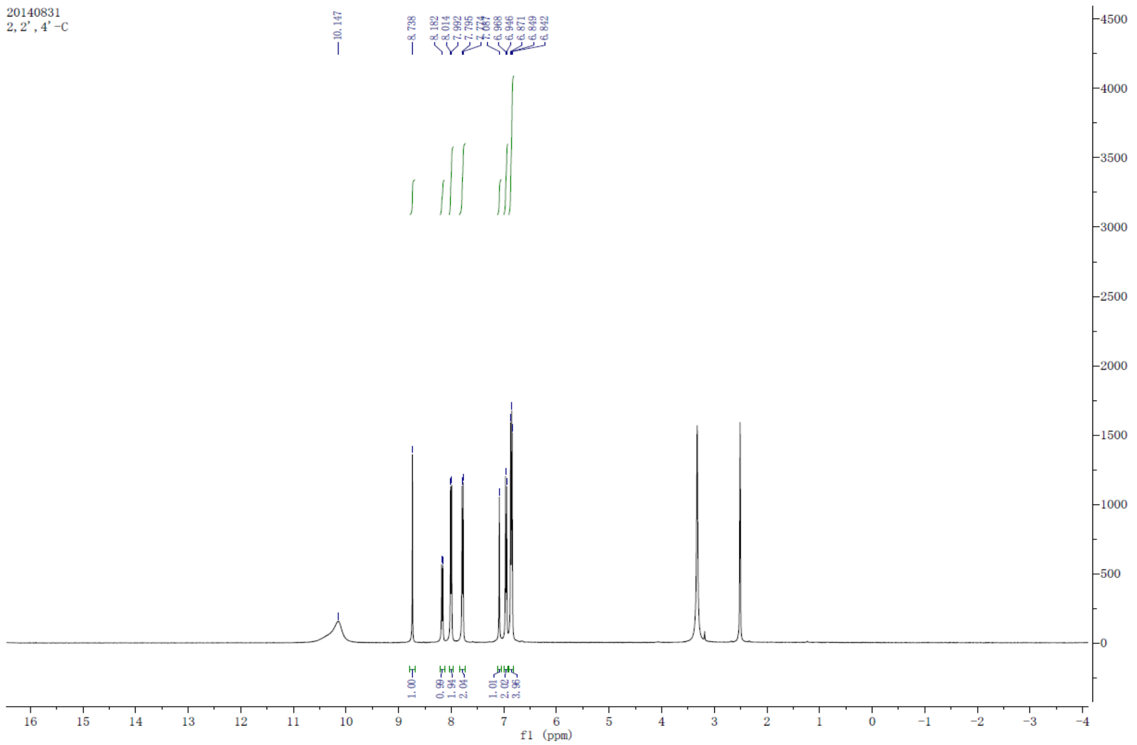

Figure S15. ^{1}H NMR of 2-[7-hydroxy-2-(4-hydroxy-phenyl)-chromen-4-ylidene]-1-(4-hydroxy-phenyl)-ethanone (**4d**)

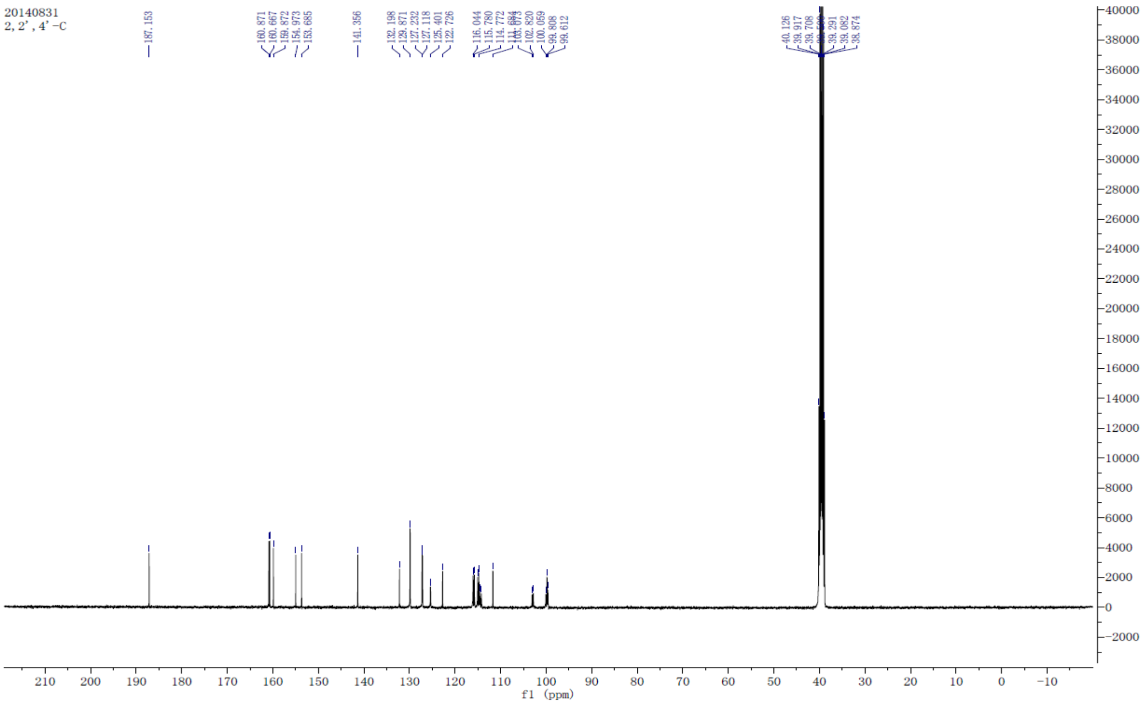

Figure S16. ^{13}C NMR of 2-[7-hydroxy-2-(4-hydroxy-phenyl)-chromen-4-ylidene]-1-(4-hydroxy-phenyl)-ethanone (**4d**)

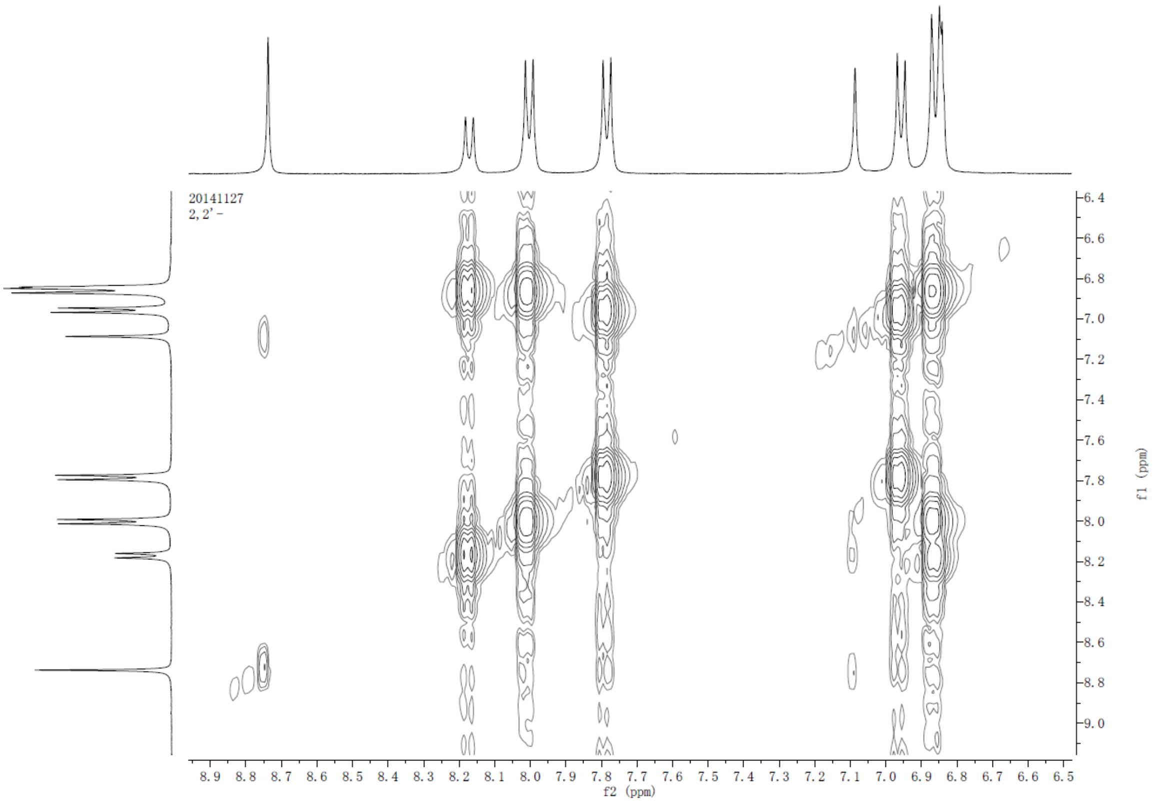

Figure S17. ^{1}H-^{1}H COSY of 2-[7-hydroxy-2-(4-hydroxy-phenyl)-chromen-4-ylidene]-1-(4-hydroxy-phenyl)-ethanone (**4d**)

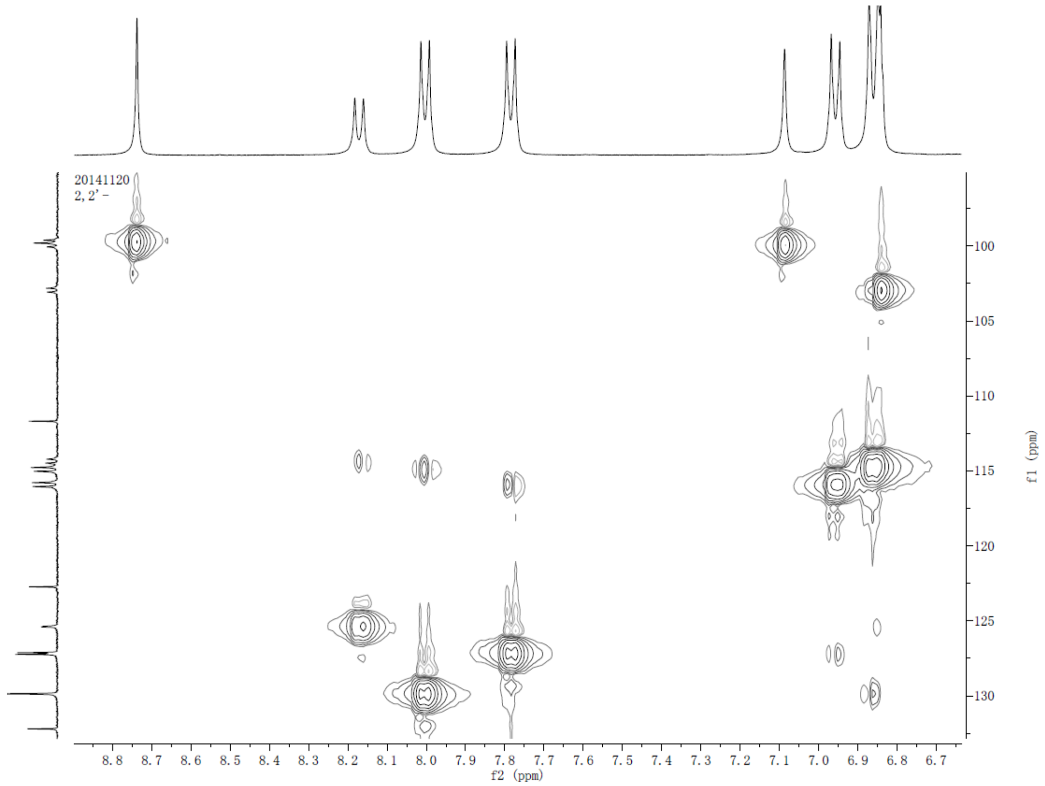

Figure S18. HSQC of 2-[7-hydroxy-2-(4-hydroxy-phenyl)-chromen-4-ylidene]-1-(4-hydroxy-phenyl)-ethanone (**4d**)

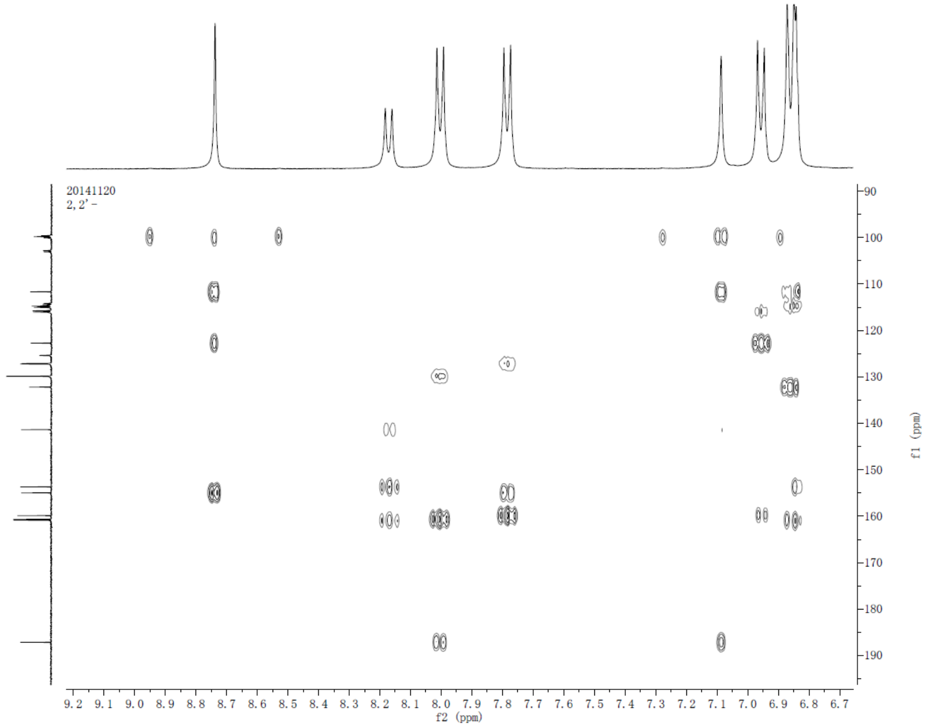

Figure S19. HMBC of 2-[7-hydroxy-2-(4-hydroxy-phenyl)-chromen-4-ylidene]-1-(4-hydroxy-phenyl)-ethanone (**4d**)

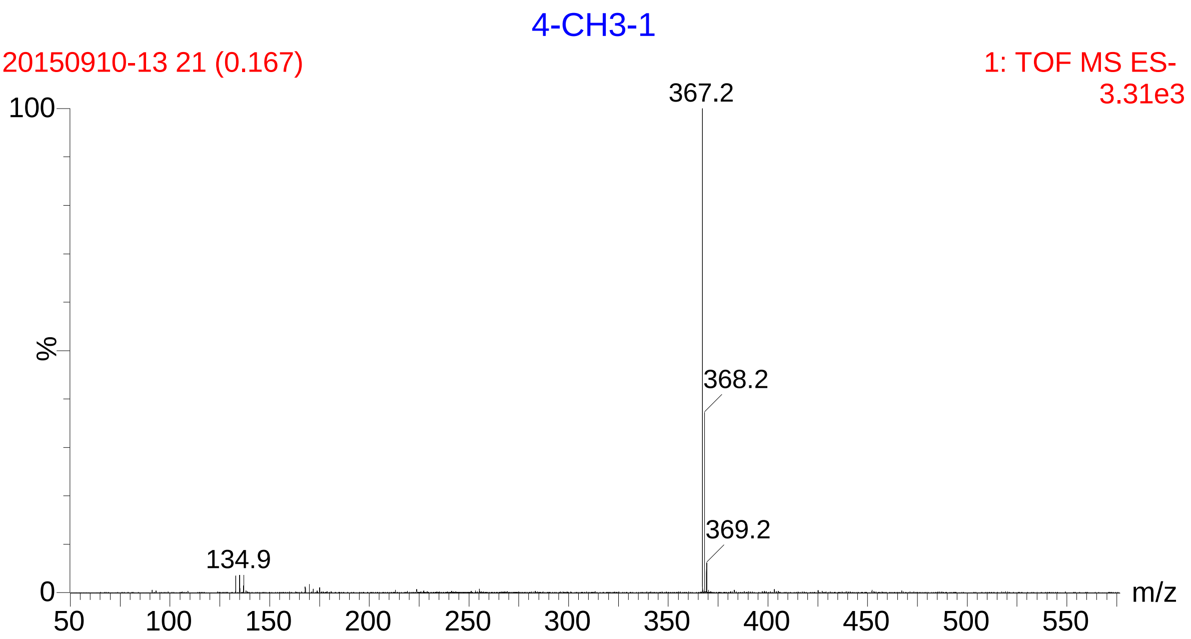

Figure S20. ESI-MS of 2-(7-Hydroxy-2-p-tolyl-chromen-4-ylidene)-1-p-tolyl-ethanone (**4e**)

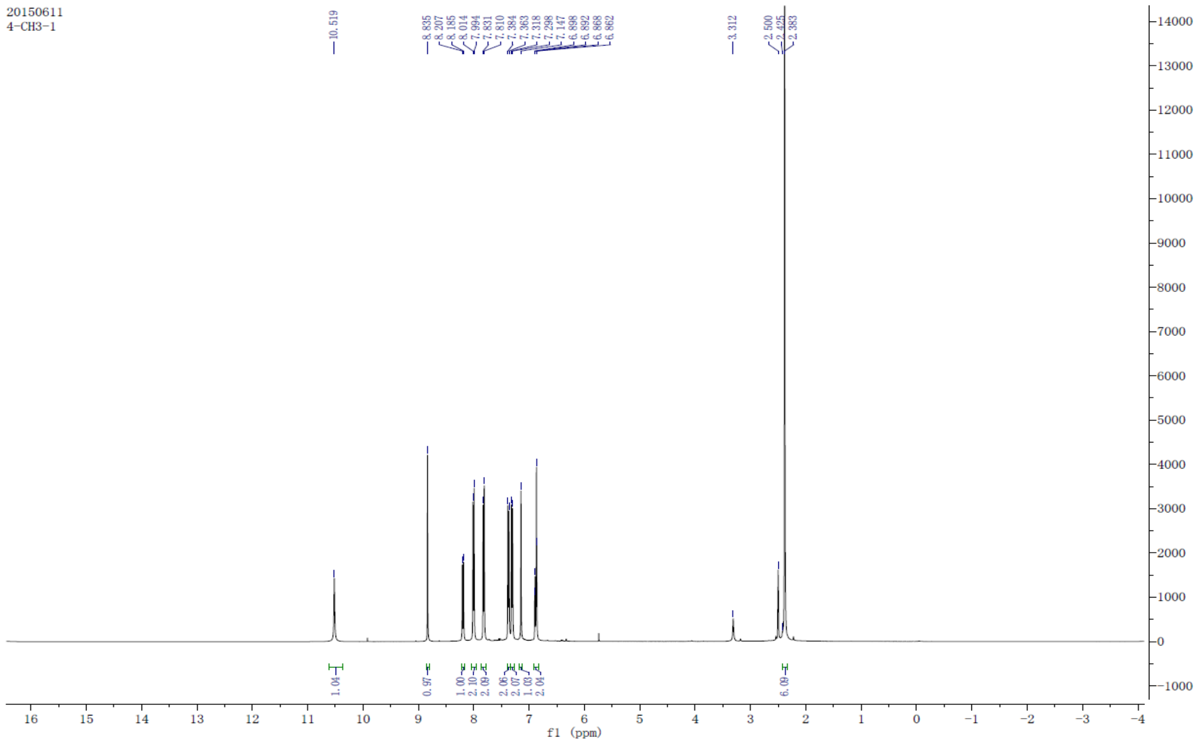

Figure S21. ^{1}H NMR of 2-(7-Hydroxy-2-p-tolyl-chromen-4-ylidene)-1-p-tolyl-ethanone (**4e**)

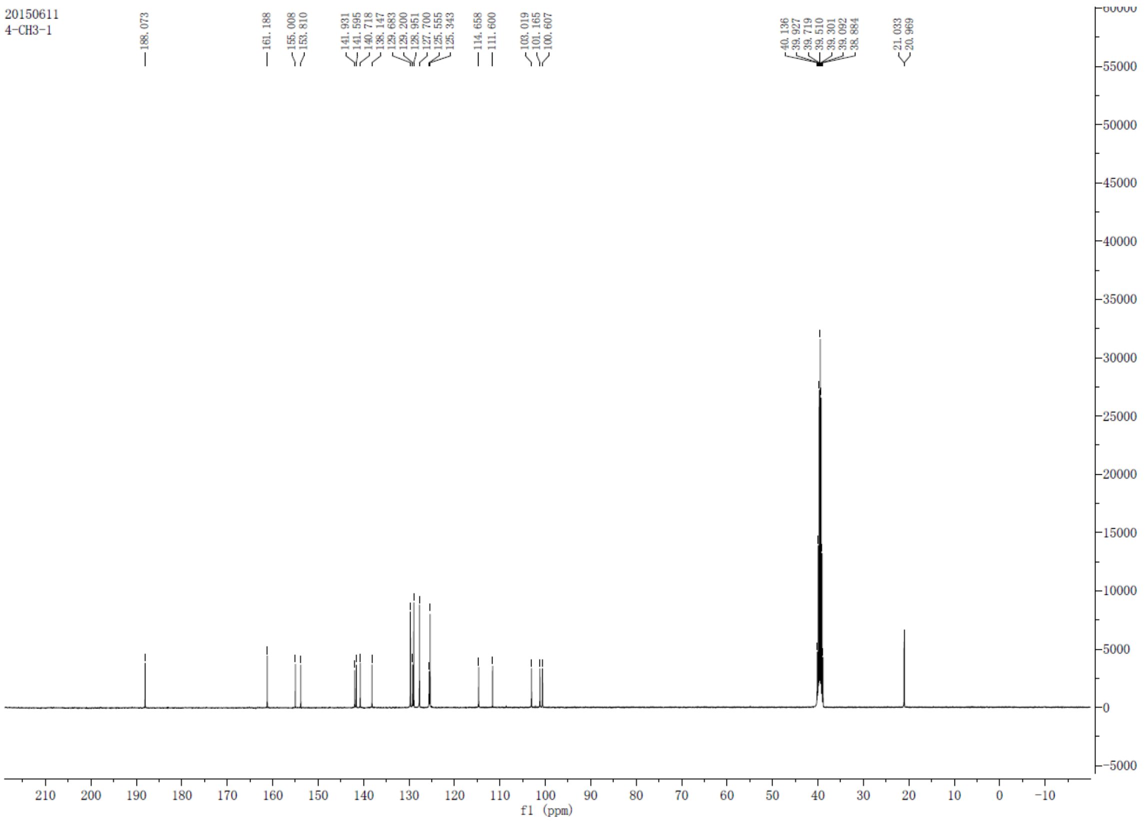

Figure S22. ^{13}C NMR of 2-(7-Hydroxy-2-p-tolyl-chromen-4-ylidene)-1-p-tolyl-ethanone (**4e**)

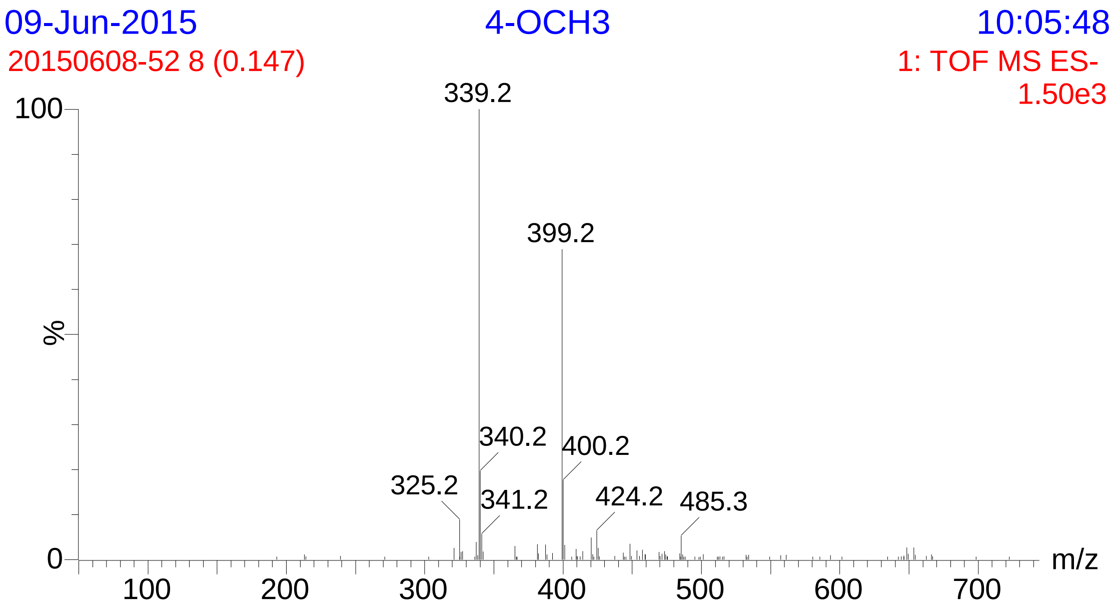

Figure S23. ESI-MS of 2-[7-hydroxy-2-(4-methoxy-phenyl)-chromen-4-ylidene]-1-(4-methoxy-phenyl)-ethanone (**4f**)

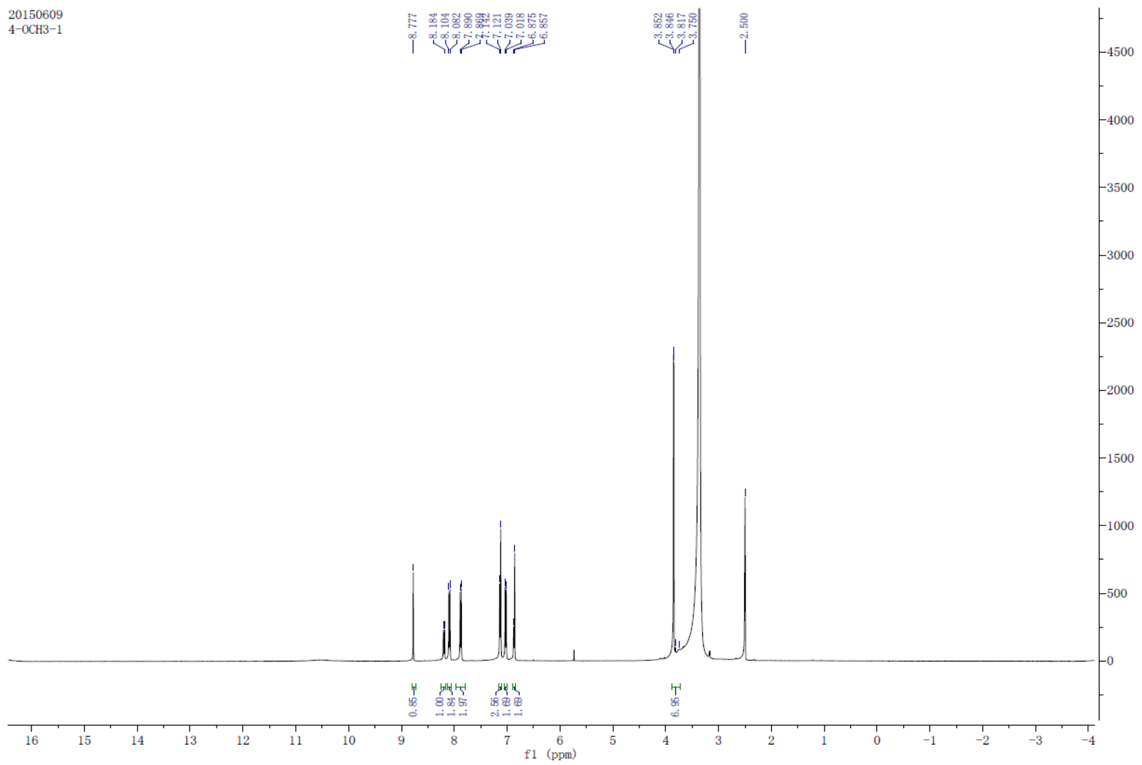

Figure S24. ^{1}H NMR of 2-[7-hydroxy-2-(4-methoxy-phenyl)-chromen-4-ylidene]-1-(4-methoxy-phenyl)-ethanone (**4f**)

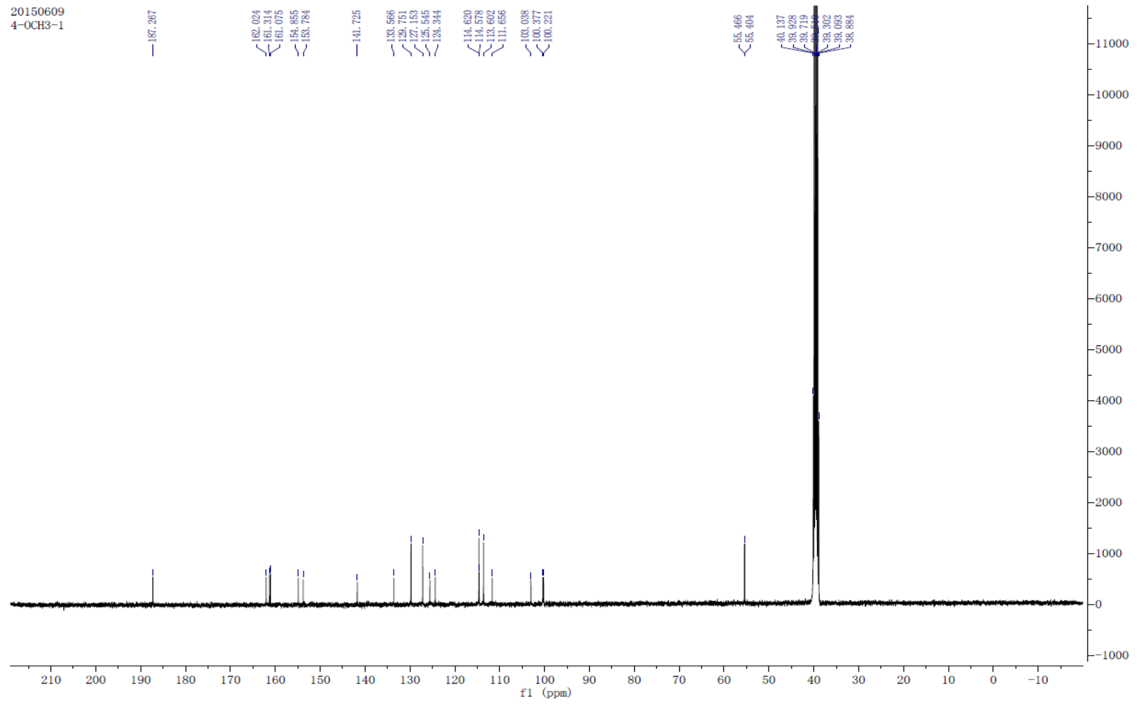

Figure S25. ^{13}C NMR of
2-[7-hydroxy-2-(4-methoxy-phenyl)-chromen-4-ylidene]-1-(4-methoxy- phenyl)-ethanone (**4f**)

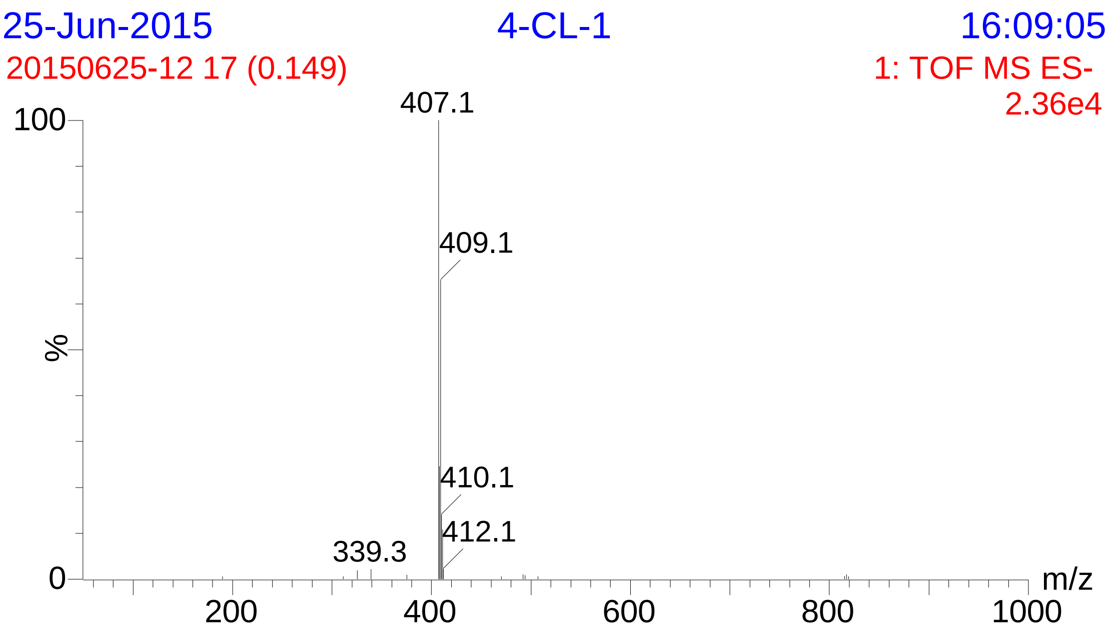

Figure S26. ESI-MS of 1-(4-Chloro-phenyl)-2-[2-(4-chloro-phenyl)-7-hydroxy-chromen-4-ylidene]-ethanone (**4g**)

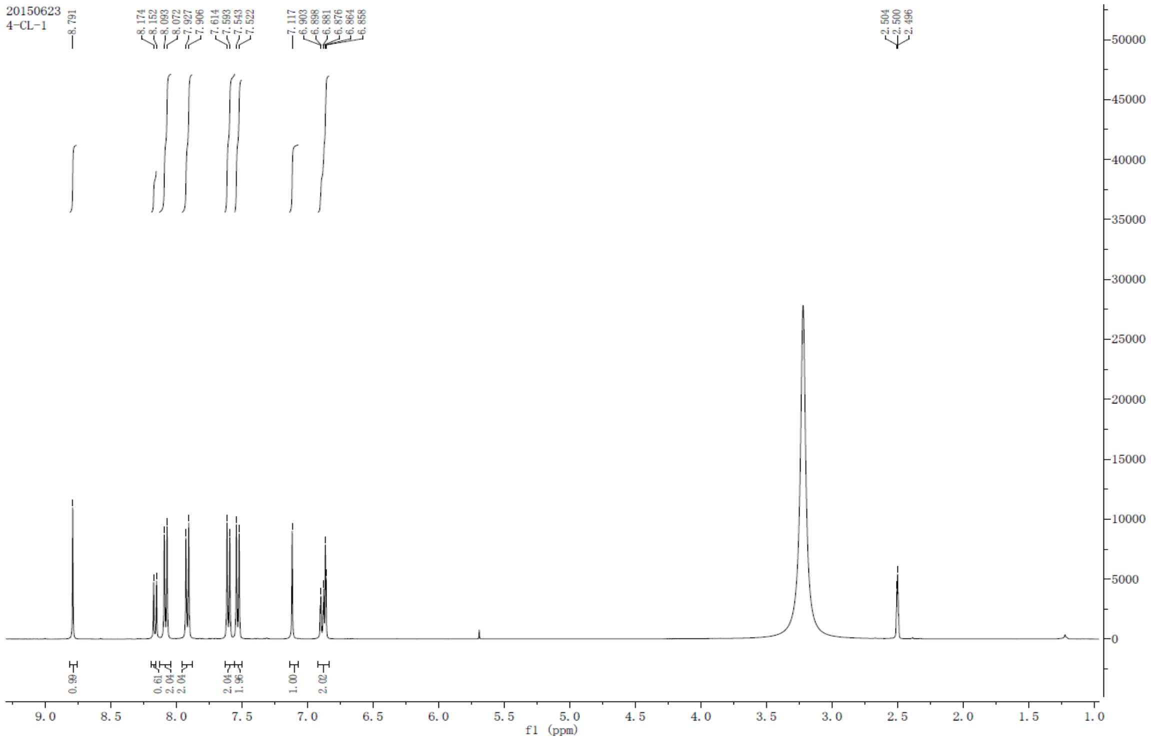

Figure S27. ^{1}H NMR of 1-(4-Chloro-phenyl)-2-[2-(4-chloro-phenyl)-7-hydroxy-chromen-4-ylidene]-ethanone (**4g**)

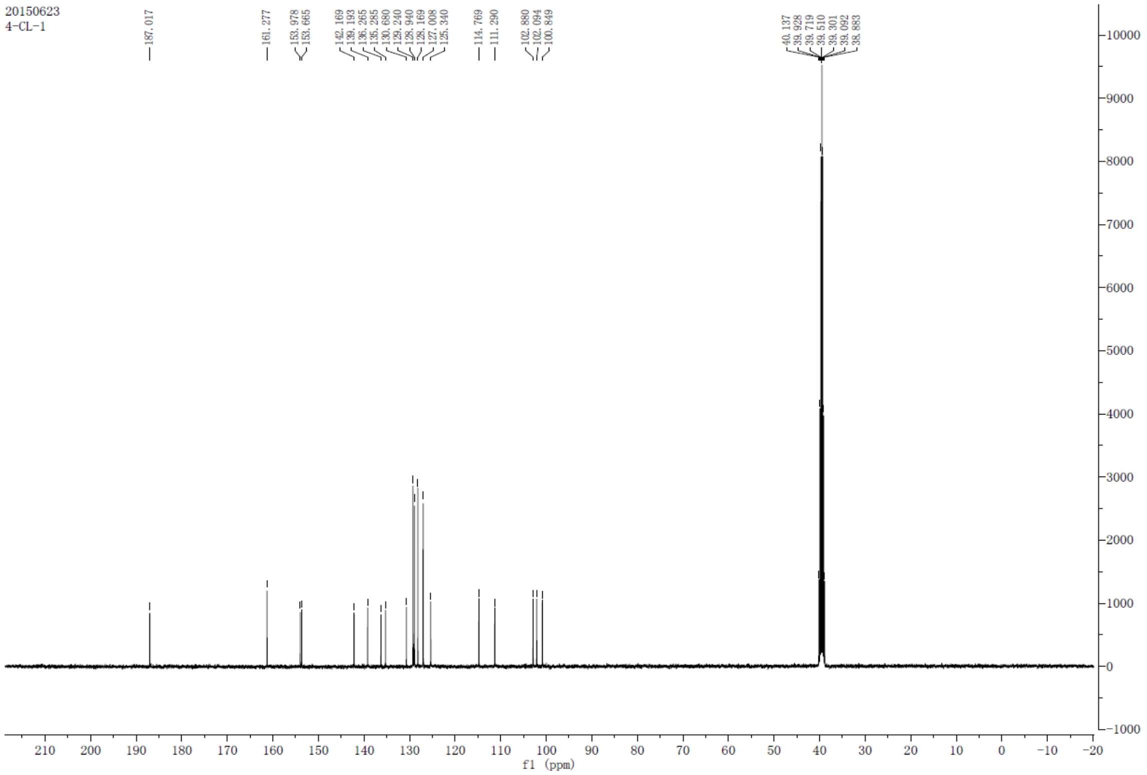

Figure S28. ^{13}C NMR of 1-(4-Chloro-phenyl)-2-[2-(4-chloro-phenyl)-7-hydroxy-chromen-4-ylidene]-ethanone (**4g**)

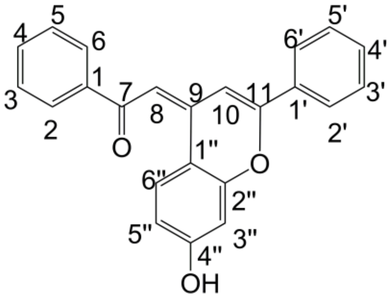

4a

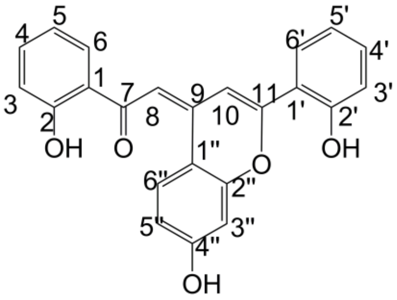

4b

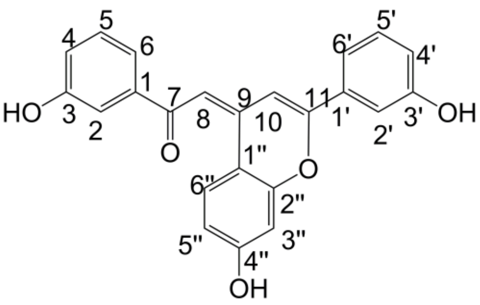

4c

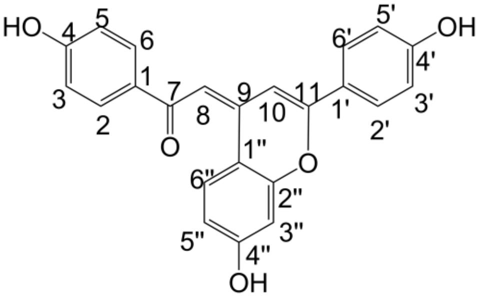

4d

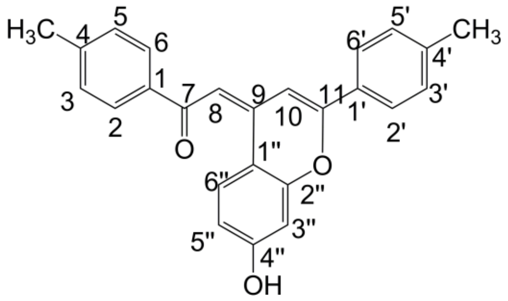

4e

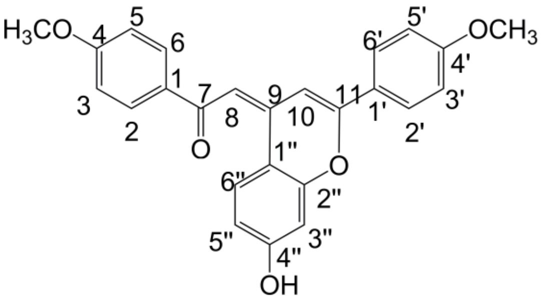

4f

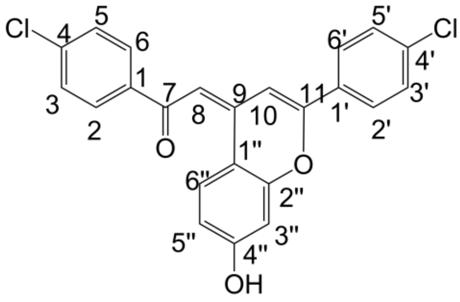

4g

Figure S29. The structures of compounds **4a-4g**
